# Alpha-Helical Protein KfrC Acts as a Switch between the Lateral and Vertical Modes of Dissemination of Broad-Host-Range RA3 Plasmid from IncU (IncP-6) Incompatibility Group

**DOI:** 10.3390/ijms22094880

**Published:** 2021-05-05

**Authors:** Monika Mitura, Ewa Lewicka, Jolanta Godziszewska, Malgorzata Adamczyk, Grazyna Jagura-Burdzy

**Affiliations:** 1 Laboratory of DNA Segregation and Cell Cycle of Proteobacteria, Institute of Biochemistry and Biophysics, Polish Academy of Sciences, 02-106 Warsaw, Poland; mmitura@ibb.waw.pl (M.M.); e.sloniewska@ibb.waw.pl (E.L.); godziszewskaj@gmail.com (J.G.); 2 Faculty of Chemistry, Chair of Drug and Cosmetics Biotechnology, Warsaw University of Technology, 00-664 Warsaw, Poland; madamczyk@ch.pw.edu.pl

**Keywords:** alpha-helical KfrC protein, broad-host-range RA3 plasmid, IncU (IncP-6) group, active partition, conjugative transfer

## Abstract

KfrC proteins are encoded by the conjugative broad-host-range plasmids that also encode alpha-helical filament-forming KfrA proteins as exemplified by the RA3 plasmid from the IncU incompatibility group. The RA3 variants impaired in *kfrA*, *kfrC*, or both affected the host’s growth and demonstrated the altered stability in a species-specific manner. In a search for partners of the alpha-helical KfrC protein, the host’s membrane proteins and four RA3-encoded proteins were found, including the filamentous KfrA protein, segrosome protein KorB, and the T4SS proteins, the coupling protein VirD4 and ATPase VirB4. The C-terminal, 112-residue dimerization domain of KfrC was involved in the interactions with KorB, the master player of the active partition, and VirD4, a key component of the conjugative transfer process. In *Pseudomonas putida*, but not in *Escherichia coli*, the lack of KfrC decreased the stability but improved the transfer ability. We showed that KfrC and KfrA were involved in the plasmid maintenance and conjugative transfer and that KfrC may play a species-dependent role of a switch between vertical and horizontal modes of RA3 spreading.

## 1. Introduction

The existence of a complex filamentous network called the cytoskeleton that spatially organizes the content of a cell has long been regarded as typical for eukaryotic cells. About 30 years ago, the bacterial cell division tubulin-like protein FtsZ, which was able to self-assemble into fibers, was discovered. It initiated the identification of various prokaryotic cytoskeletal proteins that are homologous to all three major types of filament-forming proteins comprising the eukaryotic cytoskeleton: actin (e.g., MreB, FtsA, MamK, and Alp), tubulin (e.g., FtsZ, TubZ, and PhuZ) and intermediate filament IF (e.g., crescentin) [1,2,3]. These proteins have been shown to fulfill pivotal cellular functions (reviewed in [4,5]) such as cell wall synthesis, maintenance of a cell’s shape, cell division, as well as DNA segregation and organization of intracellular components [6]. Besides the canonical cytoskeletal proteins, a variety of filament-forming proteins found in bacteria have no eukaryotic homologs, highlighting the complexity of the bacterial cytoskeleton. Among them are the Walker A Cytoskeletal ATPases (WACAs), a widely distributed subfamily of the P-loop NTPases that form ATP-dependent filaments involved in DNA segregation and cell division [7], and bactofilins performing a range of different cytoskeletal tasks [7,8]. Furthermore, there is a growing group of coiled-coil-rich proteins (CCRPs) that are putatively able to polymerize into filamentous structures in a nucleotide-independent manner mediated by the coiled-coils but lacking in typical features of eukaryotic IF proteins. They are considered as a component of the bacterial cytoskeleton or to play an auxiliary function, but, so far, they have not been investigated as extensively as the aforementioned proteins [5,9,10].

Prokaryotic cytoskeletal proteins have been found encoded not only chromosomally but also by phages [11,12] and the low-copy-number plasmids from different incompatibility groups in which they convey the function of plasmid DNA segregation [7]. Noticeably, the conjugative plasmids of the IncP, IncU, IncW, and PromA groups that are able to replicate, be stably maintained, and efficiently disseminate in a broad range of hosts encode the alpha-helical, coiled-coil-containing, DNA-binding proteins, designated KfrAs [13,14,15,16,17,18,19,20]. It has been recently shown that the presence of KfrAs is widely spread in various species [18]. At least for the IncP and IncU homologues, it was shown that filament-forming KfrAs play an accessory function in the proper plasmid segregation [13,14,15,16,17,18,19,20]. In the same two groups of the BHR conjugative plasmids, KfrAs are accompanied by presumably alpha-helical proteins, designated KfrCs, with which KfrAs interact [13,18,21]. The questions how they act and what exactly their auxiliary role is in plasmid segregation remain unanswered.

The object of our research, conjugative broad-host-range RA3 plasmid, an archetype of the IncU incompatibility group (designated IncP-6 in *Pseudomonas* spp), is a unit-copy replicon of 45.9 kb (GeneBank Accession no. DQ401103) that is able to transfer and be maintained in *Alpha*-, *Beta*- and *Gammaproteobacteria* [22]. The RA3 backbone genome contains the clusters of functionally related genes, designated the replication, the stability, and the conjugation modules.

**Figure 1:**
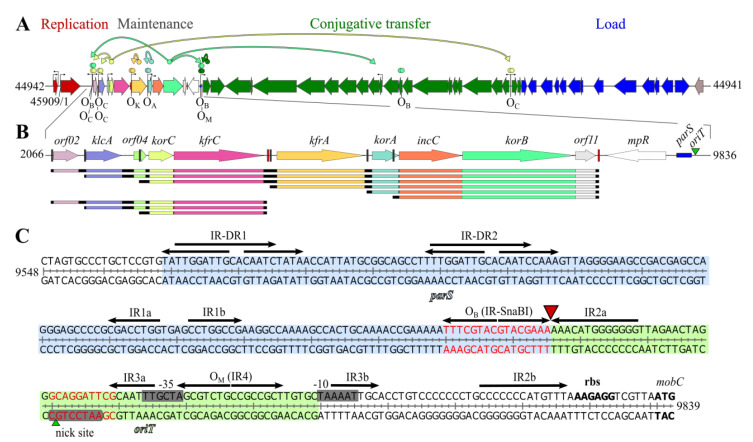
Genetic organization of RA3 plasmid from IncU incompatibility group (GenBank: DQ401103.1). (**A**) Linear map of RA3 plasmid. The replication module is in red, stability module is multicolored (close-up in Panel B), the conjugative module in green, and integron in blue. ORFs are represented by thick arrows that point out the direction of transcription. Thin black arrows in the backbone fragment indicate the transcription start sites (TSS). The colored arrows connecting the regulatory genes with the action sites of their products demonstrate the regulatory circuits (O_A_-operator for KorA, O_B_ for KorB, O_C_ for KorC, O_K_ for KfrA, and O_M_ for MobC). (**B**) RA3 maintenance module with the identified variants of the transcripts for particular genes [26]. Black boxes indicate promoters and red boxes depict Rho-independent transcriptional terminator sites. A *cis*-acting site in partition, *parS*, marked as a blue rectangle, is located in the vicinity of the origin of conjugative transfer *oriT*, marked as a green triangle. (**C**) DNA sequence of RA3 *parS*/*oriT* region located at the border of the maintenance and conjugative transfer modules. Direct motifs (DR) and arms of inverted repeats (IR) are depicted by arrows. The centromere-like *parS* region encompassing the binding site O_B_ (IR-SnaBI) for partitioning protein KorB preceded by IR-DRs is highlighted in blue [24]. The *oriT* region located between O_B_ and including O_M_ (operator for MobC) overlaps *mobCp* (grey boxes) and is highlighted in green. The conserved *nick* motif is circled in grey with a green triangle indicating a relaxase nicking site. The ribosome binding site (rbs) and start codon for MobC are in bold. The *parS* and *oriT* sequences deleted in RA3 mutants are denoted in red whereas the site of DNA insertion to separate *parS* and *oriT* motifs is pointed out by a red triangle.

The backbone functions are regulated by several autoregulators [23,24] and two global regulators, KorB and KorC, coordinating all plasmid functions (Figure 1A) [17,25]. An additional regulatory mechanism detected during analysis of the RA3 stability module expression is based on the transcriptional organization of this module and adjusts the particular gene transcript dosage to the various hosts (Figure 1B) [26]. Two important *cis*-acting sites, *parS,* the centromere-like site of the active partition system, and *oriT*, the origin of the conjugative transfer, are adjacent in the RA3 genome (Figure 1C) and may impose a steric hindrance between segrosome [24] and relaxosome complexes [27]. Within the stabilization module, upstream of the type Ia active partition system encoding KorB of the ParB family and IncC, the Walker-type ATPase of the ParA family, there are two structural genes for alpha-helical proteins KfrA and KfrC [22,28]. It has been shown recently that KfrA acts as a transcriptional autoregulator and is able to form long filamentous structures in the presence of plasmids carrying its cognate binding site [18,29]. Moreover, it forms a complex with KfrC and with both active partitioning proteins, KorB and IncC [18]. The highly unstable test plasmid that was stabilized by the presence of the RA3 stability module displayed the increased segregation rate in some hosts when deprived of the *kfrA* operon [26,29] and/or *kfrC* [18]. To better understand the functions the Kfr proteins play in the RA3 plasmid biology, the present study focused on the detailed analysis of the KfrC properties and on a wide-range search for KfrC partners among the plasmid RA3- and host-encoded proteins.

Using this approach, we identified the conjugative coupling protein VirD4 as the KfrC_RA3_ partner and mapped their interaction domains. The interplay between the conjugative transfer and the active segregation processes was demonstrated. KfrC plays an important species-dependent role in the switch between the horizontal and vertical spreading of RA3.

## 2. Results

### 2.1. Role of KfrA and KfrC in the Stable Maintenance of RA3 Derivatives in Various Hosts

Previous studies on KfrA_RA3_ and KfrC_RA3_ roles in the stability of the low-copy-number plasmid were conducted with the use of the test vector pESB36 based on the RK2 minireplicon [26]. The very unstable pESB36 was efficiently stabilized by the presence of the *orf02-orf11* RA3 stability module in the *E. coli* strain as well as in the other tested hosts but to a various extent. Deletion of either kfrs from the module or even substitution of WT *kfrA* by the mutated allele *kfrA_L43A_*, producing KfrA unable to bind specifically to DNA, led to the high plasmid instability in *E. coli* [18] and in other hosts. It seemed important to follow the effects of kfrs deletions on maintaining of the whole RA3 plasmid, i.e., in the presence of the immanent replication system and the conjugative transfer module.

**Figure 2:**
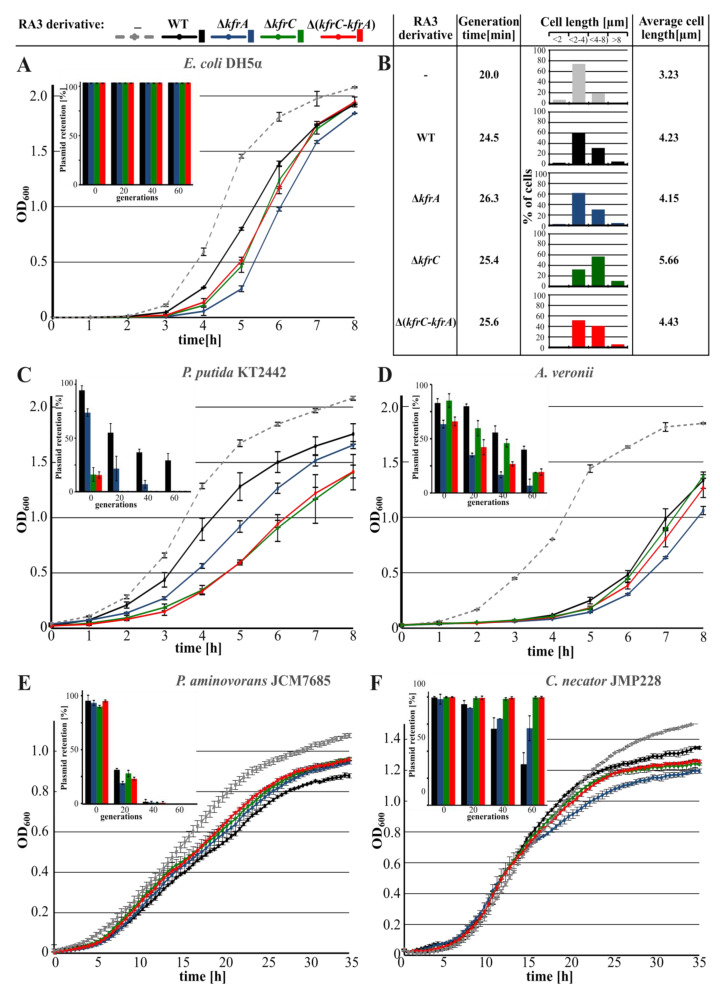
RA3 deletion variants in various hosts. (**A**) Growth of *E. coli* DH5α strain transformed with various RA3 derivatives. The inset demonstrates the results of the stability experiment carried on in triplicates. Plasmid retention was analyzed during 60 generations of growth without selection, estimated every 20 generations as the % of antibiotic-resistant colonies. (**B**) Generation time of the DH5α transformants was calculated based on the colony forming units (c.f.u.) at different time points. Microscopic observations of DAPI-stained cells were the basis for the cell size profiling and calculation of the average cell length. (**C**) Growth and plasmid stability (inset) of the RA3 transconjugants of *P. putida* KT2442 strain. (**D**) Growth and plasmid stability (inset) of the RA3 transconjugants of *A. veronii* strain. (**E**) Growth and plasmid stability of the RA3 transconjugants of *P. aminovorans* JCM7685 strain. (**F**) Growth and plasmid stability of the RA3 transconjugants of *C. necator* JMP228 strain. Transformants and transconjugants were grown in L broth at the appropriate temperature and streptomycin concentration. Broken lines represent growth curves of the plasmid-less hosts grown without antibiotic. The presented results are representative of three experiments and show average from three biological repeats (cultures grown in parallel) with standard deviation.

Deletion mutants of RA3, RA3Δ*kfrA*, RA3Δ*kfrC*, and RA3Δ(*kfrC*-*kfrA*), were constructed by replacing particular ORF(s) with the Km^r^ cassette [30]. For each RA3 deletion derivative, the growth rate and the stability functions were determined in the various hosts. The presence of WT RA3 decreased the growth rate by 25% and clearly increased the number of filamentous cells (> 4 µm) from 19% in DH5α to 37% in DH5α(RA3) given the average cell length elevated by 31% (Figure 2A,B). Deletion of the *kfrC* gene potentiated the effect of filamentation shifting the number of cells longer than 4 µm up to 70% and the average cell length about 75% in comparison to DH5α (from 3.23 µm to 5.66 µm). Hence, the presence of RA3 deprived of *kfrC* disturbs cell division, leading to the further filamentation and the longer generation time (Figure 2B). Notably, monitoring the plasmid retention during approximately 60 generations of growth without a selection demonstrated loss of neither of the four plasmids in the *E. coli* host (inset in Figure 2A).

WT RA3 and three deletion mutants were introduced via conjugation into two other strains of *Gammaproteobacteria*, *Pseudomonas putida* and *Aeromonas veronii,* and the representative strains of *Alpha*- and *Betaproteobacteria*, *Paracoccus aminovorans* and *Cupriavidus necator*, respectively. The growth of *P. putida* KT2442, similarly to DH5α, was retarded in the presence of RA3 plasmid (Figure 2C). While removal of KfrA increased generation time from 41 min to 44 min, the presence of RA3Δ*kfrC* and RA3Δ(*kfrC*-*kfrA*) extended generation time to 49 min. Here, the results were in-line with the decreased retention of RA3 derivatives. RA3Δ*kfrA* segregated slower than the other two variants, RA3Δ*kfrC* and RA3Δ(*kfrC*-*kfrA*), which were lost from the population after only 20 generations of growth without selection (inset in Figure 2C).

In *A. veronii*, only the presence of RA3Δ*kfrA* increased the generation time by 5% despite that all three deletion derivatives were less stable than WT RA3. Among them, RA3Δ*kfrA* segregated quicker than the two other mutants did (Figure 2D).

No clear difference in the growth rate and stability was observed between transformants carrying WT RA3 and its three derivatives in *P. aminovorans* of *Alphaproteobacteria* (Figure 2E).

In *C. necator* of *Betaproteobacteria*, seemingly no effect on growth rate was observed in transconjugants of all three derivatives in comparison to the WT RA3 transconjugant (Figure 2F). Stability experiments, however, demonstrated increased retention of the RA3 variants with deletions in the *kfr* genes in comparison to WT RA3 (inset in Figure 2F), suggesting the negative interference of Kfrs in the stable maintenance of RA3 in this host.

### 2.2. KfrC_RA3_ Structure

**Figure 3:**
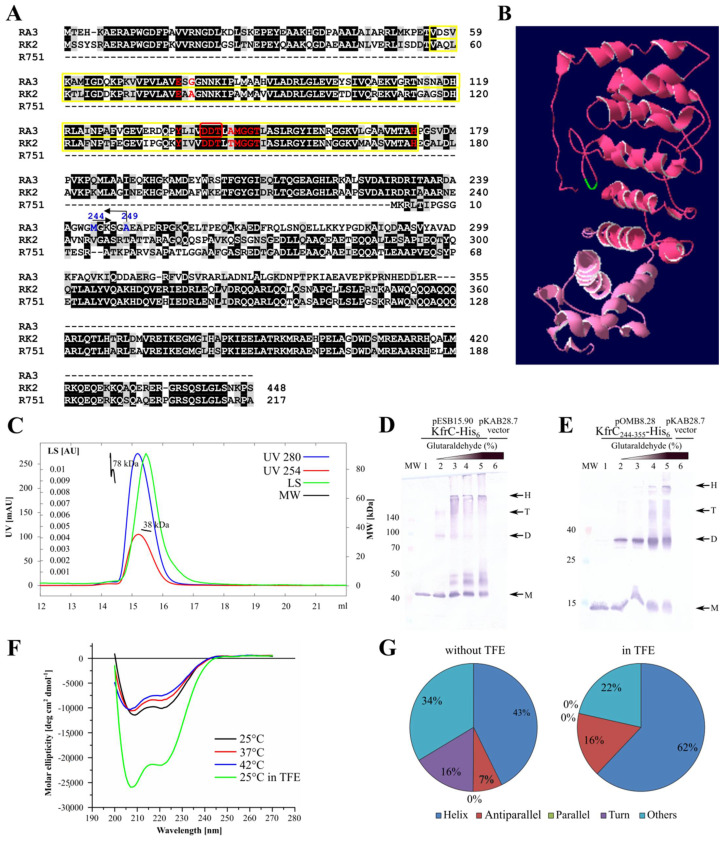
KfrC_RA3_ structure analysis. (**A**) Alignment of the closest homologs of KfrC_RA3_ (IncU) [ABD64834.1], KfrC_RK2_ (IncP-1α) [CAJ85732.1], and KfrC_R751_ (IncP-1β) [AAC64416.1]. Identical residues are shadowed in black, similar in grey. Phosphoribosyltransferase (PRT)-type I domain (Pfam: PF00156) is encircled yellow, putative active sites indicated with red font. The KfrC_RA3_ residues substituted by alanine are encircled red. Residues in blue indicate the ends of the KfrC_RA3_ truncations. (**B**) Structural KfrC_RA3_ model predicted by I-TASSER [32]. N-terminal region is highlighted in dark pink, C-terminal region in light pink. The KfrC_RA3_ residues substituted by alanine are indicated in green. (**C**) SEC-MALS analysis. The column was equilibrated with 50 mM NaPi buffer (pH 7.5), 0.15 M NaCl and KfrC-His_6_ tagged protein was dissolved in the same buffer at the final concentration of 1 mg mL^−1^. The chromatograms display curves for the light scattering (LS) and UV readings at 280 nm and 254 nm, in green, blue, and red, respectively. The scale for the LS detector is shown on the left-hand axis. The black lines (MW) indicate the calculated mass of the eluted protein (scale on the left-hand axis). The predicted molecular mass of KfrC-His_6_ monomer is 40.11 kDa. (**D**,**E**) In vivo crosslinking of the tagged KfrC-His_6_ and KfrC_244-355_-His_6_ proteins. The cell extracts of BL21(DE3) transformants containing overproduced proteins were used in the crosslinking reactions with different concentrations of glutaraldehyde. The predicted molecular mass of KfrC_244-355_-His_6_ monomer is 14.48 kDa. Complexes were separated by SDS-PAGE and analyzed by Western blotting using anti-His_6_ antibodies. Arrowheads indicate detected signals for monomers (M), dimers (D), tetramers (T), as well as the higher molecular aggregates (H). Lane MW—molecular weight marker [kDa], lanes 1–5—increasing concentrations of glutaraldehyde: 0%, 0.001%, 0.002%, 0.005%, and 0.01%, respectively. The extract of BL21(DE3) strain containing pKAB28.7 (*T7p-his_6_*) was used as a control with 0.01% glutaraldehyde (lane 6). (**F**) Far-UV circular dichroism spectra. The CD spectra were measured at various temperatures, and with the addition of TFE at a temperature of 25 °C. (**G**) The secondary structures estimated with the BestSel program [33] for KfrC_RA3_ with or without the addition of TFE at a temperature of 25 °C are presented.

KfrC of RA3 (355 amino acids) is homologous (68% identity) in the first 240 residues to the N-terminal part of KfrC (448 amino acids) from RK2 (IncPα). Interestingly, this N-terminus of KfrC_RK2_ is deleted in the representatives of IncPβ, e.g., R751 (Figure 3A). Since the remaining part of KfrC_RA3_ (115 amino acids) has no homologs in the database, the *kfrC*_RA3_ gene was split accordingly into two fragments (encoding 1–249 and 244–355 amino acids) to find out their potential functions. The predicted model of KfrC_RA3_ by I-TASSER is shown in Figure 3B, with two domains differently colored. The N-terminal domain not only contains conserved 5-phosphoribosyl-1-pyrophosphate (PRPP) binding motif (V56-H173) (Figure 3A) but also has the characteristic fold of phosphoribosyltransferase (PRT)-type I domain (Pfam: PF00156).

The model of KfrC_RA3_ by I-TASSER predicted the high content of alpha-helices (Figure 3B). To verify it, the *kfrC* gene was cloned into pET28 derivative (pESB15.90) and the KfrC-His_6_ protein was purified by affinity chromatography. It was shown that C-terminally His_6_-tagged KfrC (pOMB9.29), when over-produced in *E. coli,* retained the properties of the intact KfrC (see the next section).

The purified KfrC demonstrated the dominance of the monomeric form (40 kDa) in solution as shown via SEC-MALS analysis (Figure 3C). The KfrC potential to dimerize was tested during in vitro experiments by the use of the cross-linking agent glutaraldehyde (GA). After cross-linking the extracts of induced BL21(DE3) pESB15.90 (*kfrC-his_6_*) or pOMB8.28 (*kfrC_244-355_*-*his_6_*), transformants were separated by PAGE and KfrC was visualized by Western blotting with anti-His tag antibodies. The extract from BL21(DE3) pKAB28.7 (empty vector) was also treated with 0.1% glutaraldehyde and used as a control (Figure 3D,E). The ability of KfrC to form dimers and the higher-order complexes was demonstrated. A similar spectrum of complexes was observed after cross-linking of KfrC_244-355_, pointing out this part of KfrC as the dimerization domain.

The secondary structure of KfrC was analyzed using the circular dichroism method (Figure 3F,G). It confirmed the alpha-helical structure of KfrC in the range of temperatures between 25 °C and 42 °C. Addition of TFE (2,2,2-trifluoroethanol) [31] promoted the stability of the molecules, increasing the estimated alpha-helix content from 43% to 62%.

### 2.3. Inhibition of Hosts’ Growth by the Abundance of Kfr Proteins

**Figure 4:**
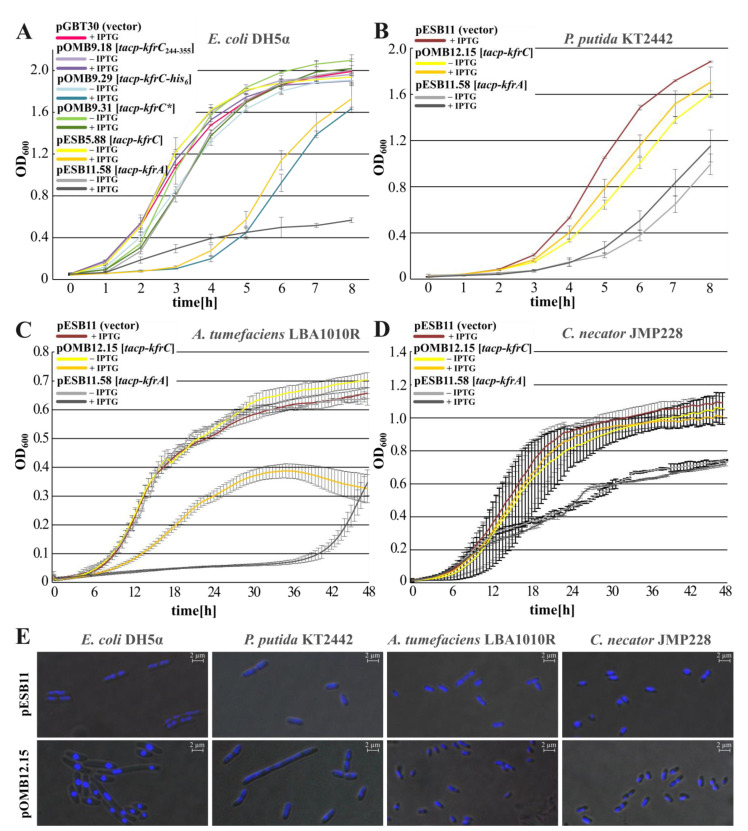
Overproduction of Kfr proteins. Transformants and transconjugants were grown in the selective L broth with and without 0.5 mM IPTG at the appropriate temperature. The presented results are representative of three experiments and show the average from three biological repeats (cultures grown in parallel) with standard deviation. (**A**) Effects of KfrA and KfrC variants abundance in *E. coli* DH5α strain. (**B**) Effects of KfrA and KfrC abundance in *P. putida* KT2442, (**C**) in *A. tumefaciens* LBA1010R, and (**D**) in *C. necator* JMP228. (**E**) Microscopic observations of DAPI-stained transconjugants cells carrying either an empty vector pESB11 or pOMB12.15 overproducing KfrC. Images were intensified when required.

The WT *kfrC* gene and the 3′ *kfrC* fragment encoding KfrC_244-355_ were cloned into the high-copy-number expression vector pGBT30 and overproduced in the *E. coli* DH5α strain. Overexpression of the intact *kfrC* from pESB5.88 caused significant retardation of the host growth (Figure 4A) as overproduction of KfrC-His_6_ did (pOMB9.29). The abundance of the C-terminal fragment of KfrC_RA3_ (pOMB9.18) did not affect the bacteria growth whereas attempts to clone the *kfrC_1-249_* under *tacp* into pGBT30 led to the various plasmid DNA rearrangements. Since the N-terminal part of KfrC_RA3_ was predicted to encode a putative phosphoribosyltransferase, it was decided to modify the postulated enzymatic center (DDT motif at positions 141–143, Figure 3A) by the triple alanine substitutions. The clone was stable and the variant designated KfrC * when overproduced (pOMB9.31) did not cause growth retardation of the *E. coli* DH5α transformant (Figure 4A). This suggests that the toxicity of KfrC might be related to its putative enzymatic activity.

To analyze the KfrC overproduction effect in other RA3 hosts, the *kfrC* was re-cloned under control of *tacp* into mobilizable pESB11, the modified BHR vector to obtain pOMB12.15. The excess of KfrC on the host growth was tested in *P. putida*, another representative of *Gammaproteobacteria*, *Agrobacterium tumefaciens,* the representative of *Alphaproteobacteria*, and *C. necator* of *Betaproteobacteria*. The KfrC “toxicity” was clearly host-dependent. Strong growth inhibition was observed in *A. tumefaciens* (Figure 4C), the weaker inhibition in *P. putida* (Figure 4B), and no effect of KfrC overproduction was observed in *C. necator* (Figure 4D).

Microscopic observations of various hosts cells carrying pESB11 (vector) or pOMB12.15 (*tacp-kfrC*), grown in the presence of 0.5 mM IPTG, revealed strong condensation of the nucleoids in the presence of KfrC excess in *P. putida* and *E. coli*. Weaker condensation effects were observed in *A. tumefaciens* and *C. necator* (Figure 4E). The species-characteristic reactions on the excess of KfrC, e.g., growth retardation, nucleoids condensations, suggested variability of KfrC targets in these hosts. Since KfrC forms a complex with the KfrA [18], the effects of KfrA overproduction were also analyzed after mobilization of pESB11.58 (*tacp*-*kfrA*) to the various strains. The KfrA excess affected growth of all tested hosts much stronger than the excess of KfrC did (Figure 4A–D).

### 2.4. Mapping of the KfrC Domain of Self-Interactions and Interactions with KfrA and KorB

Previous BACTH analysis [18] showed that KfrC and KfrA strongly interacted with each other. The ability to form a complex between these two alpha-helical proteins was also confirmed by coimmunoprecipitation experiments. The domain of interactions between KfrA and KfrC was mapped to the long alpha-helical tail (KfrA_54__–__355_) with the fragment KfrA_54–177_ exhibiting much stronger association with KfrC than KfrA_178–355_ [18]. In this work, mapping of the KfrC domain of self-interactions and interactions with the previously identified partners, KfrA and KorB, was undertaken.

**Figure 5:**
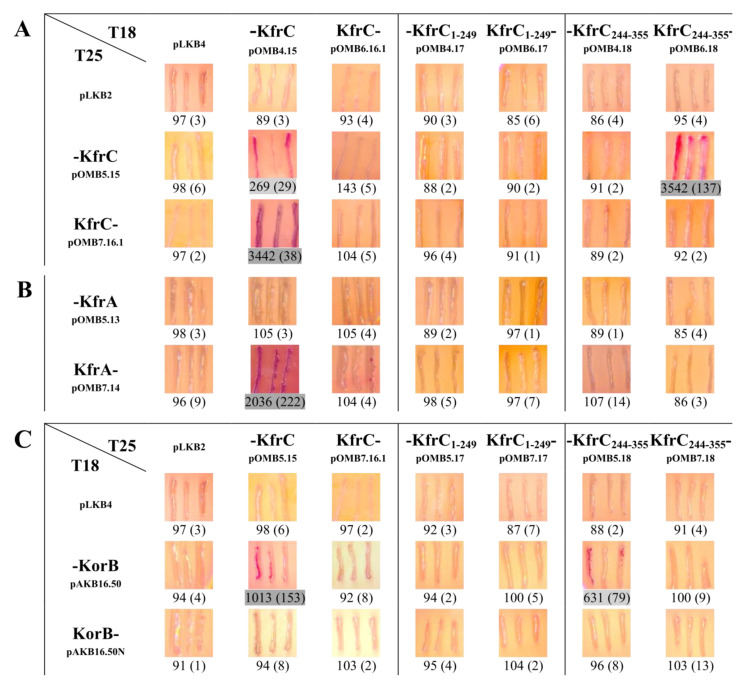
Mapping of the KfrC_RA3_ domains using the BACTH system. Analysis of domains involved in the homodimerization (**A**) and heterodimerization with KfrA (**B**) and KorB (**C**). Double transformants of *E. coli* BTH101 with compatible plasmids encoding CyaA fragment T18 or T25 fused to the analyzed proteins from N- or C-terminus were tested on indicator MacConkey plates with maltose as a carbon source and by β-galactosidase assays in the liquid cultures. Dark (purple) streaks are indicative of interactions between the two hybrid proteins. Numbers below the images represent β-galactosidase units from at least three experiments with SD in brackets. Dark grey and light grey shadings indicate strong and weak interactions, respectively. Double transformants with one empty BACTH vector versus vector encoding the full-length protein were used as the controls (the first column).

Two gene fragments (*kfrC_1__–__249_*, *kfrC_244__–__355_*) and the intact *kfrC* were cloned into the four vectors of BACTH [34] system facilitating translational fusions with the CyaA fragments from N- or C-termini. Re-constitution of the CyaA activity as a result of interactions between the hybrid proteins leads to the expression of sugar catabolic genes such as *mal* or *lac* operons. The ability to interact was tested on indicator MacConkey plates with maltose and activity of β-galactosidase was assayed in the liquid cultures of the *E. coli* BTH101 *cyaA* transformants. The KfrC dimerization domain was mapped to the C-terminal 112 amino acids KfrC_244__–__355_ (Figure 5A).

Splitting KfrC into two parts abolished strong interactions between KfrA and KfrC proteins, suggesting that either the intact KfrC was required to form a complex with KfrA or the genetic manipulation impaired the interaction domain in KfrC_RA3_ (Figure 5B).

Previously, it was demonstrated that both components of the Kfr complex had the ability to interact with the RA3 segrosome proteins and KfrA interacted strongly with KorB (ParB homolog) and weakly with IncC (ParA homolog), whereas KfrC interacted only with KorB [18]. Here, we mapped the domain of interactions with KorB to the C-terminal dimerization part of KfrC, KfrC_244__–__355_ (Figure 5C).

### 2.5. Search for the KfrC_RA3_ Partners

A wide genomic approach was undertaken to search for putative partner proteins encoded in the *E. coli* and *A. veronii* genomes since *Aeromonas* spp are the most widely spread RA3 hosts in the aquatic environments [35]. The high-quality (>95% inserts) genomic libraries of these two organisms (producing “prey” polypeptides) were prepared in the high-copy-number BACTH vector pUT18C [34] (Figure S1). The “bait” proteins, CyaA_T25_-KfrC (pOMB5.15) or KfrC-CyaA_T25_ (pOMB7.16.1), were produced in the BTH101 transformants. Selection of the possible interactants was conducted by plating BTH101 double transformants on the minimal medium with maltose as a carbon source, antibiotics, X-gal, and IPTG added to follow simultaneously the expression of the *lac* operon. Plasmid DNA isolated from the chosen “positive” clones was used to transform BTH101 with the appropriate bait plasmid using the same medium. Two-step screening allowed us to diminish the pool of the “false positives”. Plasmid DNA isolated from the chosen clones was sequenced. The results of this search are presented in Table 1.

Screening of both libraries from *E. coli* and *A. veronii* identified mainly membrane-associated proteins and several enzymes engaged in the phosphometabolism. Since clones in the libraries encoded the fragments of the structural genes, it was necessary to validate the results by cloning complete ORFs into the BACTH system. The vast majority of the analyzed ORFs lost the ability to interact with KfrC in the plate tests, although there were a few that sustained this activity (Figure S2). Further studies are required to establish the functional connections between KfrC and these proteins.

Another approach was taken to identify the KfrC frontline partners, besides KfrA, among proteins encoded by the RA3 plasmid (Table 2). The genomic library of RA3 was prepared in the same high-copy-number pUT18C vector and screened in the same way as the bacterial genomic libraries. The identified partners were KfrC itself (3 clones), VirB4 (2 clones), and VirD4 (17 clones). Both VirB4 and VirD4 presumably have the ATPases activities and are components of the T4SS (Type IV secretion system) involved in the RA3 conjugation. During conjugation VirD4, the coupling protein (CP), is assumed to deliver the relaxosome complex of relaxase bound at *oriT* with the single-stranded plasmid DNA to a membrane-associated transferosome complex. VirB4 is a part of a transmembrane channel interacting directly with the CP and participating in the relaxosome secretion [36].

**Figure 6:**
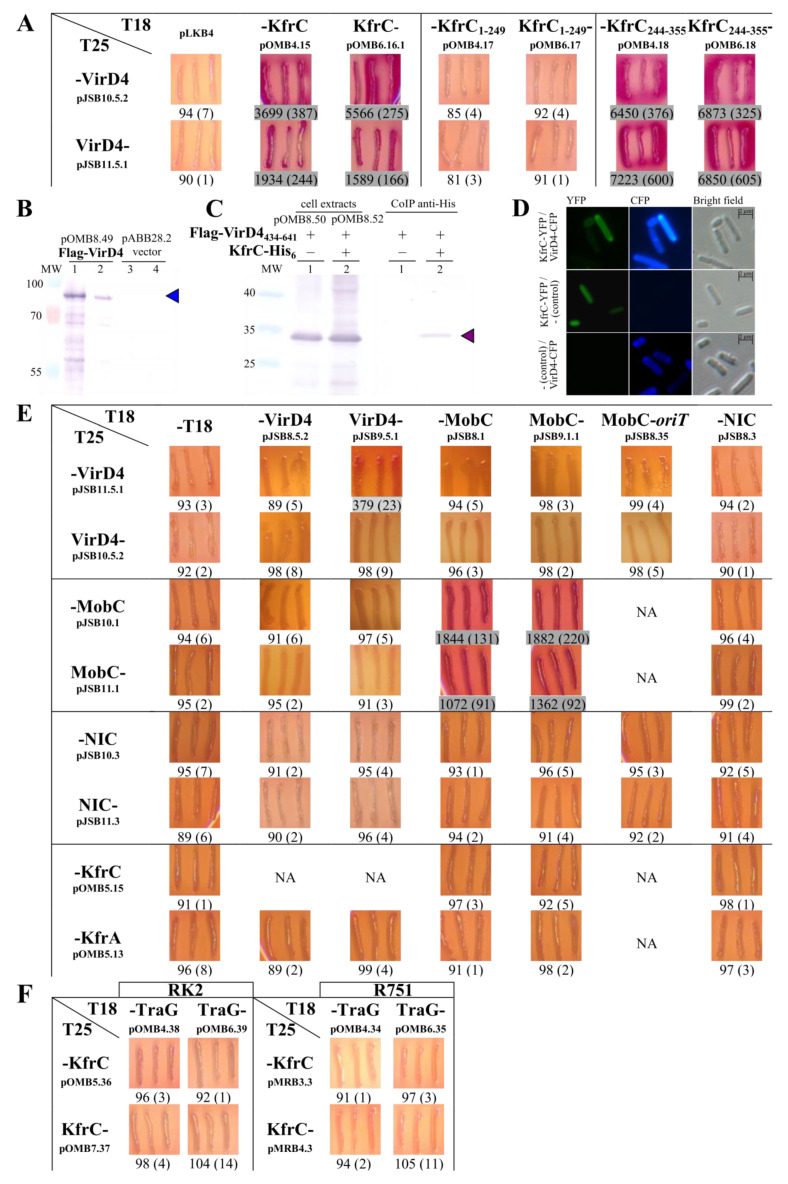
KfrC_RA3_ interactions with the conjugative coupling protein VirD4 and with the relaxosome proteins. (**A**) Mapping of the VirD4_RA3_ interaction domain within KfrC_RA3_. The detailed description as in Figure 5. (**B**) Overproduction of FLAG-tagged VirD4_RA3_ (arrowhead) analyzed by SDS-PAGE and Western blotting with anti-FLAG antibodies. Lane 1 and 2—the cell debris and the soluble fraction of *E. coli* BL21(DE3) (pOMB8.49) extract, respectively. Lane 3 and 4—the cell debris and the soluble fraction of *E. coli* BL21(DE3) (pABB28.2) extract, respectively. (**C**) Immunoprecipitation of complexes between KfrC_RA3_ and VirD4_434–641_. FLAG-VirD4_434–641_ was overproduced in BL21(DE3) either from pOMB8.50 (*T7p*-*flag-virD4_434–641_*) or together with KfrC-His_6_ from pOMB8.52 (*T7p-flag-virD4_434–641_-kfrC-his_6_*). After immunoprecipitation with anti-His antibodies, proteins were separated by PAGE and screened with anti-FLAG antibodies in the Western blot procedure. Initial cellular extracts (left), proteins immunoprecipitated with the use of anti-His antibodies (right). Arrowhead, FLAG-VirD4_434–641_ (26 kDa). Lane MW—molecular weight markers [kDa]. (**D**) Colocalization of KfrC_RA3_-YFP (pAKB2.70) and VirD4_RA3_-CFP (pOMB12.74) in *E. coli* DH5α cells assayed by the fluorescence microscopy. Images were taken with the use of the appropriate filters for the two proteins in question. Bright field images served as the controls. (**E**) Interactions between RA3 relaxosome proteins NIC and MobC, the coupling protein VirD4, and Kfr proteins. The detailed description as in Figure 5. NA, not assayed in this set of tests. (**F**) Interactions between homologs of KfrC and VirD4 (TraG) of IncP plasmids, RK2 (IncPα), and R751 (IncPβ). Reciprocal plasmid combinations with TraG fusion proteins produced from the low-copy-number pKT25 and KfrC from pUT18 derivatives gave the same negative results.

Fourteen of the 22 “positive” clones of the RA3 library contained variable-length C-termini of VirD4. It confirmed that the main interaction domain was inherent to the last 46 residues of VirD4 (Table 2). The three clones fished out with CyaA_T25_-KfrC demonstrated interactions with a central part of VirD4 (244–283 residues), which suggested the possibility of two VirD4 domains of interactions with KfrC. To verify the interactions of the full ORF, *virD4* was cloned into the BACTH system and strong interactions between KfrC and VirD4 were demonstrated. It was also shown that the C-terminal part of KfrC_RA3_ is engaged in the interactions with VirD4 (Figure 6A).

Attempts to demonstrate interactions between full-length FLAG-VirD4 and KfrC-His_6_ in the extracts of BL21(DE3) transformants by Co-IP were unsuccessful because VirD4 was found in the cell debris fraction after sonication (Figure 6B). Since the N-terminal part of VirD4 contains a putative transmembrane domain, it was decided to tag only the C-terminal part—VirD4_434-641_. The KfrC–VirD4 interactions were then confirmed by Co-IP between KfrC-His_6_ and FLAG-VirD4_434__–__641_ (Figure 6C). FLAG-VirD_434__–__641_ was detected in the precipitate obtained with the use of anti-His antibodies. Finally, it was decided to see whether putative partners colocalize in a cell. Both KfrC and VirD4 were fluorescently labelled as KfrC-YFP and VirD4-CFP. Proteins were produced from the compatible expression vectors, pAKB2.70 and pOMB12.74, respectively, and introduced to the *E. coli* DH5α strain separately or together (Figure 6D). KfrC-YFP gave a dispersed signal in the cells in the absence of the partner whereas VirD4-CFP formed bright foci at the poles in the majority of the cells in the presence and absence of KfrC. Notably, KfrC-YFP also formed foci close to the poles when VirD4-CFP was present in the cells, implicating that its polar positioning depended on VirD4.

As it was mentioned above (Figure 3A), the closest homolog of KfrC_RA3_ is KfrC_RK2_ of the IncPα plasmid [37]. However, the similarity concerns only the first 240 residues, which are lost from IncPβ representatives, e.g., KfrC of the R751 plasmid [38]. The 115-amino-acid polypeptide from the C-terminus of KfrC_RA3_ has no homologs in the database. If the acquirement of a new C-terminus by KfrC_RA3_ was the evolutionary way to accomplish a domain of interaction with VirD4, then both KfrC variants from IncPα and IncPβ plasmids (Kfr_R751_ is homologous to the C-end of KfrC_RK2_ that is not present in KfrC_RA3_, Figure 3A) should be deprived of this ability. Hence, the *kfrC* genes of RK2 and R751 were cloned into the BACTH system along with the cognate coupling proteins TraGs, homologs of VirD4_RA3_, and analyzed for interactions (Figure 6F). No interactions have been detected among KfrC and TraG proteins of RK2 and R751.

### 2.6. The Interactions between the KfrC_RA3_ Partners Involved in Two Modes of Plasmid Spreading

Previously, it was established that partitioning proteins KorB_RA3_ and IncC_RA3_ dimerize and interact with each other [17] and that an alpha-helical, filamentous KfrA protein forms a complex with KfrC and interacts with KorB and IncC [18]. KfrC also heterodimerized with KorB_RA3_. Here, we showed interactions of KfrC with VirD4, the coupling protein in the conjugative transfer process, as well as with VirB4, an ATPase participating in the inner membrane part of the transferosome. Using the BACTH system, we analyzed the interactions between the RA3 relaxosome proteins, the VirD4 protein, and the Kfr proteins. The RA3 relaxosome consists of the NIC relaxase and *oriT* [27]. Additionally, it was shown that MobC acts as an auxiliary protein potentiating the NIC cleavage at *oriT* [27]. NIC also collaborated with the MobC in the autoregulation of the *mobC-nic* operon [23].

After cloning of the *nic* and the *mobC* into the the BACTH vectors, we demonstrated that VirD4 interacted with neither NIC nor MobC and only weakly self-associated (Figure 6E). MobC, the autorepressor and the auxiliary transfer protein [23], strongly dimerized, but MobC–NIC interactions and dimerization of NIC were not detected. The presence of *oriT* did not facilitate interactions between the relaxase and the coupling protein that was observed in other conjugation systems [39,40,41]. Besides the KfrC–VirD4 interactions (Figure 6A), no associations of KfrA or KfrC and the relaxosome proteins were shown (Figure 6E). Altogether, these results suggest that the BACTH system is not the perfect tool to look at the formation of multicomponent complexes, especially if some of them act as the flexible/dynamic linkers. The interactions between the RA3 relaxosome components ought to be analyzed via other methods.

### 2.7. The Interplay between the Active Partitioning and the Conjugative Transfer Processes

**Figure 7:**
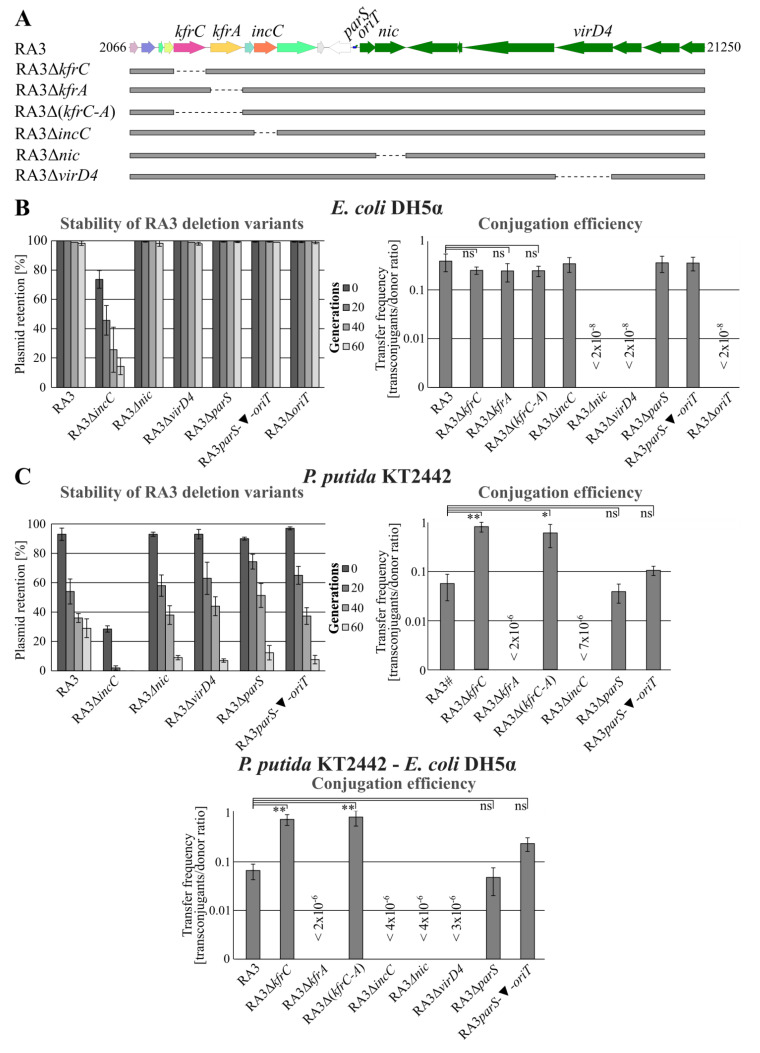
Role of KfrC_RA3_ in the plasmid stable maintenance and the efficiency of the conjugative transfer in *E. coli* and *P. putida* hosts. (**A**) Schematic presentation of RA3 variants used in these experiments. Other tested RA3 variants, RA3Δ*parS*, RA3Δ*oriT*, and RA3 *parS oriT* insertional mutant, are depicted in Figure 1C. (**B**) Retention of RA3 variants in *E. coli* DH5α strain and their conjugative transfer frequencies between *E. coli* strains. Segregation experiments were conducted for 60 generations without selection. Quantitative conjugation was done on the nitrocellulose filters and the transfer frequency was indicated on the semilogarithmic scale as the number of transconjugants per donor cell. Data represent mean ± SD from three biological replicates. The differences in the frequency of the conjugative transfer between RA3 variants are not statistically significant (ns) (*p*-value > 0.05 in Kruskal–Wallis one-way analysis of variance). (**C**) Retention of RA3 variants in *P. putida* KT2442 strain and their conjugative transfer frequency in the intra- and the interspecies spreading. RA3# plasmid contains Km^r^ cassette within integron. Introduction of RA3 conjugation-deficient variants to *P. putida* was done with the use of the helper strain *E. coli* DH5α carrying pJSB1.24 with the RA3 conjugative transfer module and *korC* gene. Data represent mean ± SD from three biological replicates. The statistically significant differences between WT RA3 and its variants with *p*-value ≤ 0.005 or < 0.05 (based on Kruskal–Wallis one-way ANOVA followed by Tukey’s test of multiple comparisons) are indicated by two or one asterisk, respectively.

The discovered interactions of KfrC, probably when complexed with KfrA, with KorB of the segrosome and the coupling protein VirD4 (or transferosome) raised a hypothesis of an interplay between mutually exclusive processes of vertical and horizontal spreading. The eight RA3 variants deprived of *kfrA*, *kfrC*, *kfrC-kfrA*, *incC*, *virD4*, *nic*, and two *cis*-acting sites, *parS* or *oriT* (as presented in Figure 1C and Figure 7A), were constructed and used in the conjugation and stability experiments.

The additional RA3 construct had an insertion of a 1 kb fragment separating *parS* and *oriT* motifs (*parS*-▼-*oriT*) to see whether close proximity of these motifs in the RA3 genome interferes with the binding of the segrosome and relaxosome complexes (Figure 1C).

All nine derivatives together with WT RA3 (control), were introduced into the *E. coli* DH5α strain and tested for stability and frequency of conjugative transfer into the DH5α Rif^r^ recipient. Stability assays showed that all tested deletion mutants except Δ*incC* were stably maintained in *E. coli* for 60 generations (Figure 7B and Figure 2A for *kfr* deletion mutants). Unexpectedly, the deletion of the *parS* region (seemingly the important *cis*-acting site in the active partition process [24]) did not influence the RA3 stability in *E. coli*. The presence of two additional KorB binding sites in the RA3 genome offers a plausible explanation of this result. Among nine RA3 deletion variants, only mutants *virD4*, *nic*, and Δ*oriT* mutants were significantly impaired in the conjugative transfer between the *E. coli* strains. Hence, no interference between conjugative transfer processes and stability functions was noticed in this host.

Different results were obtained during the analyses of the set of RA3 mutants in the *P. putida* KT2442 strain (Figure 7C and Figure 2C, inset). WT RA3 was less stably maintained in the *P. putida* host than in *E. coli* being retained after 60 generations of growth without selection only in 30% of cells. Lack of any of Kfrs strongly destabilized RA3 and led to the loss of plasmid in 80% to 100% of cells after 20 generations. Lack of *incC* also caused a loss of the RA3 deletion derivative after 20 generations. The most spectacular results were observed when the RA3 variants were tested in the conjugation experiments. In the absence of KfrA, the RA3 conjugation frequency between *P. putida* strains decreased by more than six orders of magnitude whereas the lack of KfrC alone or both Kfrs had an opposite effect, increasing the conjugation frequency by approximately 10-fold. Separation of *parS* and *oriT* via kanamycin cassette led to a statistically insignificant increase in the conjugation frequency. Finally, deletion of *incC* had a detrimental effect not only on plasmid stability but also the frequency of the horizontal transfer and/or plasmid establishment. Similar results were obtained during interspecies conjugation between *P. putida* strains used as donors and the *E. coli* DH5α strain as a recipient (bottom diagram in Figure 7C).

## 3. Discussion

**Figure 8:**
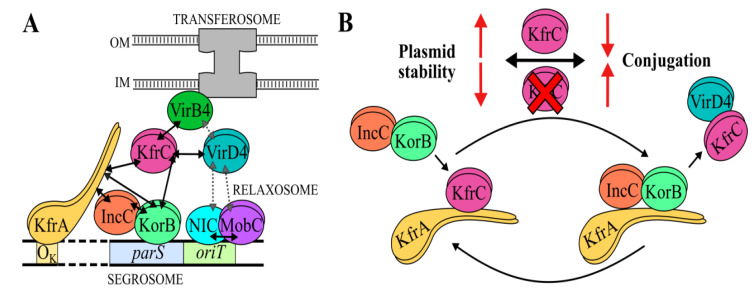
Interactions between the Kfr proteins, segrosome, relaxoxosome, and transferosome in RA3. (**A**) Model of the complexes built at *parS-oriT* region of RA3. (**B**) KfrC acts as a switch between the horizontal and the vertical spreading of RA3 plasmid. The established protein–protein interactions (this work, [17,18]) are indicated by solid arrows. Putative interactions are depicted by the broken-line arrows.

Studies on the alpha-helical KfrA protein [16] have suggested its role in the stability of IncP plasmids, together with KfrC, is encoded in the same operon [13]. It was shown that the KfrA of R751 interacted with KfrC using a linker, KfrB [13]. In the RA3 plasmid of the IncU group, KfrC could interact directly not only with KfrA but also with KorB (Figure 8A), one of the components of the active partition system [18]. It was shown that KfrA_RA3_ had the ability to form filaments [29] and interacted with both components of the partition apparatus, KorB and IncC. Hence, the accessory role of a scaffold built of Kfr complexes in the segregation of plasmid molecules to the progeny cells in a species-dependent manner was envisaged [18].

The studies on *kfrA* and *kfrC* conducted on the stability module cloned into a highly unstable heterologous replicon [18,26] proved an important role of both proteins in the stable maintenance of the test plasmid in the *E. coli*, *P. putida*, *A. tumefaciens*, and *C. necator* strains. It was also shown that the KfrA DNA binding activity was vital to support plasmid stability [18]. Here, we decided to look at the effects of the deletions of *kfrC*, *kfrA* operon, or both (Δ*kfrC-kfrA*) in the RA3 background. In *E. coli*, the presence of any of these three derivatives led to the growth retardation, a slight increase in the generation time, and, in the case of RA3Δ*kfrC*, to the formation of filamentous cells. Despite these changes, all three variants were very stably maintained for at least 60 generations of growth without selection in clear contrast to the results of the test plasmid pESB36.44 (Δ*kfrA*) based on the heterologous RK2 minireplicon [18,26]. It demonstrated that in *E. coli,* in the context of the whole RA3 genome, the Kfr proteins did not play vital roles in the segregation of RA3. The very active conjugation system or easily adaptable RA3 replication system may compensate for the difference the lack of Kfr proteins imposes on the plasmid retention. In other tested hosts, e.g., *P. putida, A. veronii*, or *C. necator*, the effects of Kfr deficiencies were much stronger not only on the growth rate but also on the stable maintenance and clearly were species-specific (Figure 2).

KfrC belongs to the alpha-helical proteins with two-domain structures. The N-terminal part with phosphoribosyltransferase (PRT)-type I domain (Pfam: PF00156) is responsible for its “toxicity” when in excess. The results of screening of *E. coli* and *A. veronii* genomic libraries strongly suggested that KfrC_RA3_ was part of the phosphometabolomes of the hosts. The metabolic role of KfrC in various hosts is under investigation. This initial libraries’ screening also revealed the interactions between KfrC_RA3_ and the various membrane-bound proteins, implicating at least temporal positioning of KfrC close to a cellular membrane. Its polar cell localization in the presence of VirD4 was demonstrated (Figure 6D).

The KfrC_RA3_ C-terminal domain of 112 residues seems to be multifaceted. Its involvement in the dimerization, in the interactions with the partition protein KorB, and the coupling protein VirD4 opens new possibilities of the KfrC role in the RA3 plasmid biology (Figure 8A).

The RA3 deletion derivatives in *kfr* genes were not only less stable than WT RA3 in *P. putida* but also demonstrated the altered conjugation frequency between *P. putida* strains and *P.-putida*- *E. coli* strains. The effect was very strong when KT2442 RA3(Δ*kfrA*) was used as a donor. The conjugation frequency was more than six orders of magnitude lower than for WT RA3. Significantly, the removal of *kfrC* or *kfrA-kfrC* stimulated 10-fold the transfer frequency of the analyzed RA3 derivatives in comparison to the WT RA3 (Figure 7C), implicating a negative role of KfrC in the conjugative transfer efficiency and a requirement for KfrA only when KfrC was present. The insignificant variation in the number of transconjugants of RA3 with the *parS* and *oriT* sites separated by a 1 kb insertion suggested that the closeness of these two important *cis*-acting sites in the WT RA3 did not affect the transfer initiation process despite the fact that they had to accommodate next to each other two large protein complexes, segrosome and relaxosome.

Our previous studies on the RA3 relaxosome demonstrated an auxiliary role of the MobC protein. The MobC binding to O_M_ in the *mobCp* had not only an autoregulatory role in the *mobC-nic* expression but it increased the nicking activity of NIC more than 1000-fold and in turn stimulated the transfer [23]. Reciprocally, the interaction of NIC with its binding site (IR3, Figure 1C) enhanced the MobC repressor action of *mobCp* [27]. The BACTH studies concerning intermolecular interactions within the RA3 relaxosome demonstrated neither NIC interactions with MobC nor with VirD4 (Figure 6E). The MobC dimerized efficiently but did not associate with VirD4 as it was observed for the auxiliary proteins in other conjugative systems [39,40,41]. Other experimental approaches are needed to elucidate the structure of the RA3 relaxosome. Hence, lack of the observed interactions between Kfr and relaxosome proteins does not exclude the possibility of their occurrence in the cells.

In this study, a new VirD4_RA3_ partner was found, the KfrC protein that somehow linked the conjugation with the active partition process not only by interactions with VirD4 but also VirB4 ATPase (Figure 8A), an important energy supplier for the transferosome [36]. VirD4s have multidomain structures [39] with (i) an N-terminal transmembrane domain responsible for the spatial positioning and interactions with the transferosome inner membrane complex (IMC), (ii) a cytoplasm facing the middle part with the ATPase domain (energy supplier), and (iii) a seven-helix motif called the all-α domain (AAD) responsible for recruiting and docking a relaxase with the covalently bound transfer DNA. The variable-length (iv) cytosolic C-terminal domains are typically enriched in the acidic residues [42,43] and are assumed to evolve to expand a range of protein effectors being transferred by T4SS as well as to control presentation of effectors to the system [39]. Notably, according to the BACTH library screening, there are two fragments of VirD4_RA3_ interacting with KfrC_RA3_: the internal hydrophobic polypeptide VirD4_244__–__283_ and the C-terminal 47 residues. Finding that VirD4 of RA3 was capable of interactions with KfrC_RA3_, whereas TraGs, VirD4 homologs of IncP plasmids, did not interact with the cognate KfrCs, correlated with VirD4_RA3_ having an extended highly acidic C-terminus in comparison to the IncP homologs (Figure S3). On the other hand, KfrC_RA3_ also differs from the IncP homologs in its C-terminal part of the 115-residue polypeptide that exhibits the high content of the polar residues (45%). This fragment, unique for KfrC_RA3_, (Figure 3A) was shown to be involved in the dimerization and binding of both KorB and VirD4.

Previously, we showed that the conjugative transfer process of RA3 was subjected to complex multilayered control mechanisms. The three conjugative transfer operons were strongly repressed by the global and the local repressors at least in *E. coli*. The *mobC-nic* operon is autoregulated by MobC and NIC [27]. The longest operon *orf33-traC3*, encoding most of the transferosome components, is regulated by the global regulator KorC in cooperation with the so far unidentified product of the transfer module [25]. The cross-talk between stability and conjugative functions is also potentiated by the fact that in this long transcriptional unit there is the internal promoter, *orf23p*, negatively controlled by the second global regulator, active partition protein KorB, bound at the distant O_B_ [17]. Divergently oriented, *orf34p* of the tricistronic operon *orf34-orf36,* is very efficiently repressed by KorC. Here, we showed that at the top of this transcriptional regulation there are protein–protein interactions, KfrC–VirD4, that decrease the efficiency of the conjugative transfer process. Our transcriptional studies conducted in different hosts showed that the highest level of gene expression in the stability module was detected for the partition operon *incC-korB-orf11* with the *korC-kfrC* operon being the second in line [26]. Constitutive expression of the *korC-kfrC* operon at the significant level may determine their importance in the control of the conjugative transfer besides its important role in the plasmid partition process.

Our model implicates that KfrC may improve the plasmid segregation by bringing the segrosome complex to the filamentous KfrA scaffold due to its ability to bind KorB and KfrA. KorB may outcompete KfrC for the KfrA binding since both proteins interact preferentially with the KfrA_54__–__177_ region [18]. The release of KfrC from the KfrA filamentous network allows it to interact with VirD4 and, in effect, to interfere with the efficient transfer of relaxosome to the transferosome at least in the *P. putida* cells. Significantly, the C-terminal dimerization domain of KfrC is involved in the interactions not only with KorB (segrosome) but also with VirD4, so it may provide a spatiotemporal switch between two processes responsible for the various modes of RA3 spreading, vertical and horizontal (Figure 8B). The strength of KorB–KfrC–VirD4 interactions may also be species-specific due to additional factors involved.

The interplay of two aspects of a plasmid physiology, stable maintenance and the conjugative expansion, was brought to the attention of researchers via the analysis of an atypical plasmid stabilization system, *stbABC*, of the conjugative BHR plasmid R388 of the IncW incompatibility group [44]. Deletion of the *stbA* encoding a DNA binding protein led to the plasmid instability and the increased transfer frequency. Oppositely, the deletion of an ATPase encoding *stbB* did not affect the plasmid maintenance, but it abolished conjugative transfer. It was postulated that the defects in both plasmid maintenance and transfer were a consequence of changes in the positioning of the *stb* plasmid mutants in the cells [44].

The correlation between the active partition and the conjugative transfer processes was also postulated for the low-copy-number conjugative R1 plasmid of the IncFII incompatibility group [45]. R1 is a narrow-host-range plasmid with the type II active partitioning system [7]. It was shown that ParM, an actin-like ATPase, interacted with TraD, a homolog of VirD4 (the coupling protein), TraC (an ATPase, a homolog of VirB4), and TraI (the relaxase). TraI also interacted with the second component of the partition system, ParR, a DNA binding protein. Importantly ParM and TraI mutually increased their enzymatic activities of NTPase and relaxase, respectively. Thus, in the case of R1, the functional collaboration of Par components with the relaxosome/transferosome complex appeared optimal for its vertical and lateral modes of dissemination.

These examples and our work indicate the importance of the integration of plasmid maintenance function with the conjugation process, although it may differ in the mechanisms, e.g., cooperation and coordination as observed for R1 and R388 or the partial exclusion as in the RA3 system. The requirements for adaptation to an environment and a host range may drive these evolutionary changes.

## 4. Materials and Methods

### 4.1. Bacterial Strains and Growth Conditions

The *E. coli* strains used were: DH5α [F^−^(*ϕ80dlacZ*Δ*M15*) *recA1 endA1 gyrA96 thi*-*1 hsdR17*(r_K_^−^ m_K_^+^) *supE44 relA1* deoR Δ(*lacZYA*-*argF*)*U196*], BL21(DE3) [F^−^ *ompT hsdS*_B_(r_B_^−^ m_B_^−^) *gal dcm* (DE3)] (Novagen), BTH101 [F^−^ *cya-99 araD139 galE15 galK16 rpsL1* (Sm^r^) *hsdR2 mcrA1 mcrB1*] [34], BW25113 [*lacI*^q^ *rrnB*_T14_ Δ*lacZ*_WJ16_ *hsdR514* Δ*araBA*-*DAH33* Δr*haBADLD78*] [30], and S17-1 [*recA pro hsdR* RP4-2-Tc::Mu-Km::Tn*7*] [46]. The rifampin-resistant mutants of *A. tumefaciens* LBA1010R [47] and *P. aminovorans* JCM7685 [48] were kindly provided by D. Bartosik, University of Warsaw, Poland, *C. necator* JMP228 was kindly provided by K. Smalla, Julius Kühn-Institut, Federal Research Institute for Cultivated Plants, Germany, and *P. putida* KT2442 was kindly provided by C.M. Thomas, University of Birmingham, United Kingdom. The spontaneous Rif^r^ mutant of *A. veronii* (kindly provided by M. Gniadkowski as an environmental *A. hydrophila* strain) was isolated in the laboratory.

Bacteria were generally grown in L broth [49] or on L agar (L broth with 1.5% *w/v* agar) at 37 °C or at 28 °C (*A*. *tumefaciens*, *C*. *necator*, *P*. *aminovorans*, *P*. *putida*, and *E*. *coli* BTH101). MacConkey agar base (BD Difco) or M9 medium supplemented with 1% maltose were used in the bacterial adenylate cyclase-based two-hybrid system (BACTH) and in the library screening, respectively [50]. If needed, media were supplemented with X-gal (5-bromo-4-chloro-3-indolyl-β-d-galactopyranoside) (40 µg mL^−1^) for blue/white screening, IPTG (isopropyl-β-d-thiogalactopyranoside) for *tacp* induction or appropriate antibiotic(s): chloramphenicol (10 µg mL^−1^ for *E*. *coli*, 50 µg mL^−1^ for *A*. *tumefaciens*, 150 µg mL^−1^ for *C*. *necator*), kanamycin (50 µg mL^−1^ for *E*. *coli*, 20 µg mL^−1^ for *P*. *aminovorans* and *P*. *putida*), tetracycline (50 μg mL^−1^ for *P. putida,* 10 μg mL^−1^ for other strains), or penicillin (sodium salt) (150 μg mL^−1^ in liquid media and 300 μg mL^−1^ for agar plates), rifampin (100 µg mL^−1^).

### 4.2. Plasmid DNA Isolation, Analysis, DNA Amplification, and Manipulation

Plasmid DNA was isolated and manipulated using standard methods [50] or kits using manufacturers’ instructions. All new plasmid constructs were verified by DNA sequencing at the Laboratory of DNA Sequencing and Oligonucleotide Synthesis, Institute of Biochemistry and Biophysics Polish Academy of Science. The list of plasmids used and constructed in this study is presented in Table 3. Oligonucleotides are listed in Table 4.

#### 4.2.1. Construction of KfrC Alanine Substitution Mutant

To introduce mutations into the putative active site of *kfrC*, a two-step PCR was used. The pairs of primers 8/13 and 12/32 (Table 4) were designed to introduce nucleotide substitutions in a particular region accompanied by the introduction of a NotI restriction site to facilitate screening. In the first step, two products were amplified on a pESB5.88 template with primers 8 and 13 or 12 and 32, which after purification served as a template in the second PCR reaction with primers 8 and 32. The final PCR product was inserted between EcoRI-SalI sites of pGBT30 to give pOMB9.31 (*tacp*-kfrC*).

#### 4.2.2. Construction of the translational fusions of FLAG with VirD_434–641_ via N-terminus and KfrC with His_6_-tag via C-terminus

The *kfrC* gene without a stop codon was amplified with primers 14/18 and cloned downstream of *virD4_434–641_* in pOMB1.42. The EcoRI-SalI fragment with both genes was re-cloned into pKAB20 and digested using MunI and XhoI restriction enzymes to create translational fusions of FLAG-VirD4_434–641_ and KfrC-His_6_, respectively. Finally, the EcoRI-SalI fragment carrying *flag-virD4_434–641_ kfrC-his_6_* was re-cloned into pKAB28 (pET28mod derivative) to obtain pOMB8.52.

#### 4.2.3. Construction of RA3# Derivative with *parS*_P1_-Km^r^ Cassette

Km^r^ cassette amplified on a pKD13 template with primers 67 and 68 and *parS*_P1_ prophage amplified on a pOMB3.104 template with primers 69 and 70 were used as a template in the second PCR reaction with primers 67 and 70. The final PCR product was inserted within integron at position 38,663 of the RA3 genome with the use of the Datsenko and Wanner method [30].

### 4.3. Bacterial Transformation and Conjugation

Bacterial transformation was done using the standard methods [50]. Electroporation was carried out using 2-mm gap cuvettes at 25 μF, 200 Ω, 2.5 kV in a Bio-Rad Gene Pulser.

The *E. coli* DH5α transformants with RA3 variants or the helper strain *E*. *coli* S17-1 harboring pESB11 or pOMB12.15 (*tacp-kfrC*) were used as the donors in the conjugations with the chosen Rif^r^ strains of *A*. *tumefaciens*, *P. aminovorans*, *A. veronii*, *C*. *necator*, or *P*. *putida* as described previously [18,26]. Briefly, aliquots of 100 µL of stationary phase cultures of the donor and recipient strains, rinsed previously with L broth, were mixed on an L agar plate and incubated overnight at 28 °C. Bacteria were washed off the plate and serial dilutions were plated on an appropriate solid medium selective for transconjugants. The frequency of the conjugative transfer of RA3 or its derivatives between *E. coli* strains or *P. putida* strains was analyzed using a modification of this method. Suspensions of donor and recipient cells were mixed on the sterile nitrocellulose filters and incubated on L agar plate at 37 °C or 28 °C. Filters were immersed into 0.2 mL of L broth, vortexed, and serial dilutions plated on L-agar with antibiotics selective for transconjugants. After 24–48 h of incubation at 28 °C or 37 °C, obtained colonies were counted. Suspension of the donor cells was treated in the same manner but incubated separately on the filter to serve as a reference. Conjugation frequency was expressed as the number of transconjugant colonies per donor colonies formed. The reported values are the average of at least three different experiments.

### 4.4. Bacterial Adenylate Cyclase Two-Hybrid (BACTH) System

Possible interactions between proteins were analyzed in vivo using the BACTH system [34] as described previously [26]. Genes encoding proteins of interest were cloned into the BACTH vectors to create translational fusions with CyaAT18 (pKGB4, pLKB4 plasmids) or CyaAT25 (pKGB5, pLKB2) fragments via N- or C-terminus, respectively. Pairs of the compatible plasmids were cotransformed into *E*. *coli* BTH101 *cyaA* and transformants were selected on L agar supplemented with kanamycin, penicillin, and 0.15 mM IPTG. Bacteria were incubated for approximately 48 h and randomly chosen transformants were re-streaked on the selective MacConkey medium with 1% maltose as a carbon source. Reconstitution of the CyaA activity due to the interactions between analyzed proteins led to the activation of sugar catabolism operons in *E*. *coli*, e.g., *mal* and *lac*, manifested by forming purple colonies on maltose containing solid medium and an increase of β-galactosidase activity in the extracts from the liquid cultures. The β-galactosidase activity was assayed using the standard method [61]. One unit of β-galactosidase is defined as the amount of enzyme needed to convert 1 μmol of *o*-nitrophenyl-β-D-galactopyranoside (ONPG) to *o*-nitrophenol and D-galactose in 1 min under standard conditions.

### 4.5. Genome-Wide Library Construction of E. coli, A. veronii, and RA3 Plasmid Using BACTH System

For genomic DNA extraction of *E. coli* DH5α and *A. veronii*, the modified method of Chen and Kuo was used [62]. For plasmid RA3, the large-scale isolation Plasmid Giga Kit (QIAGEN) was used and the additional step of electroelution of the plasmid DNA from the agarose pad into the dialysis bags was applied [63] that separated the plasmid DNA from genomic DNA contamination. Obtained DNA was fragmented and cloned into the pOMB4.0 vector as described in detail in the supplemental material. The quality of the obtained genomic library was evaluated by determination of its size, the percentage of the genome coverage, and the percentage of the plasmids that have an insert. The probability of having a particular fragment inserted in the right orientation and in frame with the *cyaT18* fragment was calculated using the formula below [64].

*p* = 1 − (1 − i/6G) ^N^

i—the mean insert size [bp]

G—the genome size [bp]

N—the number of clones obtained in the library

### 4.6. High-Throughput Screening of Interaction Partners for a Bait Protein

Genomic libraries constructed in the pUT18C vector derivative (pOMB4.0) were used for a high-throughput search of the protein–protein interactions. Between 2–60 ng of the library was transformed into 50 µL of the *E. coli* BTH101 strain harboring the vector pOMB5.15 (T25-KfrC) or pOMB7.16.1 (KfrC-T25). The transformation mixture was washed twice with 1 mL of sterile water and plated on a minimal medium with selective antibiotics, thiamine, maltose as a carbon source, X-gal as an indicator, and 0.1 mM IPTG. After 3–5 days of incubation in 28 °C, blue colonies were picked up and re-streaked on the same selective medium and grown under the same conditions. Directly from the colonies remaining blue after replating, plasmid DNA was isolated using the phenol:chloroform method [50] and obtained DNA was used to transform the *E. coli* BTH101 strain containing the bait vector and plated on selective MacConkey plates containing maltose as the sole carbon source and 0.1 mM IPTG. After 48 h at 28 °C, plasmid DNA was isolated from the clones, which appeared red. The restriction pattern analysis step allowed us to eliminate the false-positive results that arose from the genetic rearrangements within vectors and caused a reversion of Cya^−^ to Cya^+^ phenotype. After confirmation of a restriction pattern, the inserts in pOMB4.0 were sequenced and analyzed further.

Chosen isolated plasmid DNAs were used to transform the *E. coli* DH5α strain and transformants were plated on the penicillin plates and verified to be Km^S^ (cured of the bait plasmid). From a single clone (Ap^R^, Km^S^), the pUT18C derivative encoding prey protein was isolated and retested for interaction with the bait by the transformation the *E. coli* BTH101 with the bait vector or the empty pKT25 vector. Positively verified interactions resulted in PCR cloning of the DNA fragments encoding the full-length prey proteins into the BACTH system and further analysis.

### 4.7. Overexpression and Purification of His_6_-tagged Proteins by Affinity Chromatography

For overproduction, *E*. *coli* BL21(DE3) carrying pESB15.90, pET28mod derivative, encoding the C-terminally His_6_-tagged KfrC was used. The purification procedure was performed as described previously via affinity chromatography [18] with the use of a washing buffer (50 mM NaPi pH 8.0, 300 mM NaCl, 10 mM imidazole, 10% glycerol). The protein purification was monitored by SDS-PAGE using the PhastSystem (Pharmacia). Protein concentration was determined using the Bradford method [65].

For the SEC-MALS and circular dichroism methods, protein purification was performed in two steps, via affinity (Ni-NTA column, Qiagen) and gel-filtration (Superdex200 16/60 column, GE Healthcare) chromatography using an automated FPLC AKTAexpress GE system. Standard purification buffers were used with the difference that the sonication and washing buffers contained 10 mM β-mercaptoethanol.

### 4.8. Determination of Protein Oligomeric States by Size-Exclusion Chromatography Coupled to Multiangle Light Scattering (SEC-MALS)

His_6_-tagged KfrC at a concentration of 1 mg mL^−1^ purified with an automated FPLC AKTAexpress GE system was loaded on a Superdex200 10/300 GL column (GE Healthcare) equilibrated with an SEC buffer (50 mM NaPi buffer pH 7.5, 0.15 M NaCl). The protein was eluted from the column at a flow rate of 0.5 mL min^−1^. Each fraction was automatically analyzed by multiangle light scattering (DAWN HELEOS II, Wyatt Technology), UV 280/254 nm (1260 Infinity LC, Agilent Technologies), and differential refractometry (Optilab T-rEX, Wyatt Technology) detectors. Data processing and molecular mass calculations were performed with the Astra program (Wyatt Technology).

### 4.9. Crosslinking with Glutaraldehyde

The ability of a His_6_-tagged KfrC or its truncated form, KfrC_244–355_ to form dimers or multimers was examined using glutaraldehyde as described previously [66] with a slight modification. Instead of the purified proteins, 1–2 µL of cell extract containing overproduced protein was used in the crosslinking reactions. Reaction products were separated by SDS-PAGE and analyzed by Western blotting using anti-His_6_ tag antibodies. The cell extract of a strain containing an empty vector was used as a control.

### 4.10. Western Blot Analysis

The proteins separated by SDS-PAGE [50] and electrotransferred from the polyacrylamide gel to a nitrocellulose membrane (Amersham™ Protran^®^ Cytiva) using the wet transfer Bio-Rad block were subjected to immunodetection as described in the supplemental material.

### 4.11. Determination of the Protein Secondary Structure by Circular Dichroism (CD) Spectroscopy

CD measurements were carried out at 200–270 nm using a Jasco J-815 CD spectrometer with a step size of 1 nm and bandwidth of 2 nm in 1 mm path length quartz cuvettes. The protein sample purified with an automated FPLC AKTAexpress GE system at a concentration of 1.5 µM was dissolved in a 50 mM NaPi buffer (pH 7.5) containing 0.15 M NaCl with the addition of 30% TFE (2,2,2-trifluoroethanol) or without TFE. Measurements were performed at a temperature of 25 °C, 37 °C, and 42 °C, and with the addition of TFE only at a temperature of 25 °C. The partition of secondary structures was estimated with the BestSel program [33]. After subtracting appropriate blanks, the mean residue ellipticity [deg cm^2^ dmol^−1^] was calculated according to the formula below [67].

[θ]_MR_ = 100θ/(clN)

θ—ellipticity [deg]

c—the concentration of protein [M]

l—the path length [cm]

N—number of amino acids

### 4.12. Construction of RA3 Mutants Using Site-Directed Mutagenesis Based on λ Red-Mediated Recombination

Defined deletions or insertions in the RA3 plasmid were prepared with the use of the standard method [30], which allows an exchange of a genetic region by a DNA fragment conferring resistance to kanamycin with the assistance of λ Red recombinase.

DNA fragments were amplified using Phanta polymerase and primers homologous, partly (20 nt) to the vector pKD13 carrying a Km resistance cassette, and partly (50 nt) to the targeted sequence. After the DNA product digestion using the DpnI enzyme to eliminate the remaining methylated template DNA and purification, it was used for electrotransformation of electrocompetent *E. coli* BW25113 cells carrying pKD46 and RA3 plasmid prepared using a standard method with the difference that 1 mM L-arabinose was used to induce expression of λ Red recombinase. After electroporation, cell suspension was plated on selective L agar plates and incubated overnight at 37 °C, at which temperature transformant cells were cured of thermosensitive pKD46. Subsequently, the Km^r^ Ap^s^ transformants were analyzed via PCR to confirm the deletion of the desired region or proper DNA fragment insertion. For the positively verified clones, the region of deletion or insertion was amplified, and the mutant construction was confirmed via PCR product sequencing.

### 4.13. Co-Immunoprecipitation

Coimmunoprecipitation of KfrC with VirD4_434–641_ was done using a modified method previously described [68]. The *E*. *coli* BL21(DE3) strain was transformed with pOMB8.52 (*T7p*-*flag-virD4_434__–641_ kfrC-his_6_*) or pOMB8.50 (*T7p*-*flag-virD4_434__–_*_641_), which served as a control. Overnight cultures of the transformants were diluted 1:50 into a fresh medium and after one hour of growth protein overproduction was induced with 0.5 mM IPTG for another four hours. The cultures were incubated with formaldehyde (1% *v/v*) at room temperature with gentle agitation for 30 min. After sonication of the pelleted cells, the immunoprecipitation procedure proceeded with anti-His antibodies (Invitrogen) as described in the supplemental material. Western blot analysis of the initial cell extracts and the Co-IP samples with the use of anti-FLAG antibodies (Invitrogen), diluted 1:2000, was carried out after protein separation by SDS-PAGE and transfer onto a nitrocellulose membrane.

### 4.14. Determination of Growth Rate of Strains Overproducing Proteins

Overnight cultures of the analyzed strains were diluted 1:100 in L broth with IPTG (0.5 mM) or without IPTG. Cultures were grown under selective conditions at 28 °C or 37 °C with agitation for 8 h (flasks) or 24 h (VarioskanTM Lux Microplate reader) and OD_600_ measurements were performed every hour or every half hour, respectively. Growth curves were prepared based on the three cultures for each strain. The strain containing the empty vector was used as a control. Generation time, when appropriate, was estimated on the basis of colony forming units (c.f.u.) at subsequent time points of culture growth.

### 4.15. Observations of the Nucleoids after DAPI Staining

To observe nucleoid localization in the cells, an overnight culture of an appropriate strain was diluted in L broth supplemented without and with 0.5 mM IPTG when needed and grown under selective conditions with agitation to the OD_600_ 0.6–0.8. Subsequently, 200 µL of the culture were transferred to the new sterile microfuge tube and immediately mixed with the equal volume of the fixation buffer (2.68% (*w/v*) paraformaldehyde, 0.005% (*w/v*) glutaraldehyde in PBS (pH 7.4; 137 mM NaCl, 2.7 mM KCl, 10 mM Na_2_HPO_4_, 1.8 mM K_2_HPO_4_)). After 15 min of incubation at room temperature and the next 15 min incubation on ice, cells were washed twice with PBS and the pellet was re-suspended in the 200 µL of PBS. A few µL of the fixed cell suspension were placed on a microscopic slide covered with 0.01% (*w/v*) poly-l-lysine (Sigma). After 10 min of incubation, not-attached cells were removed via triple PBS washing. Microscopic slides were allowed to dry and DAPI/Vectashield mixed in proportion 1:4 was added to cover the cells (DAPI (4′, 6-diamidino-2-phenylindole) 1 μg/mL in PBS with Vectashield (Vector Laboratories Inc.)).

### 4.16. Plasmid Stability Assays

The stability of RA3 or its derivatives were tested as described previously [18]. Briefly, stationary-phase cultures grown under antibiotic selection were diluted 10^5^-fold into the fresh medium (without antibiotic) and cultivated for approximately 20 generations. In parallel, diluted cultures were plated on L agar to get approximately 100–200 colonies then 100 colonies were re-streaked onto L agar with the selective antibiotic to estimate the number of bacteria retaining the plasmid. Plasmid retention was expressed as the percentage of antibiotic-resistant colonies. Culture refreshing and plating procedures were repeated every 20 generations for up to 60 generations. For each strain, stability experiments were performed in triplicate starting from three separate colonies.

### 4.17. Fluorescence Microscopy

The strains containing appropriate expression vector derivatives encoding translational fusions of analyzed proteins with CFP or YFP under *tacp* (*lacI^q^*) were used for microscopic observations. The overnight culture was diluted 1:100 in L broth supplemented with 0.01 mM IPTG when needed and grown under selective conditions. When OD_600_ reached 0.5–0.8, the cells were washed twice with PBS and the pellet was re-suspended in the PBS and a few µL of the cell suspension were applied on a microscopic slide. Bacterial cells were visualized at 100× magnification using a fluorescent microscope Carl Zeiss Axio Imager.M2 and AxioCamMR5 camera. Collected pictures were processed and analyzed with the program AxioVision Rel.4.8.2 (Carl Zeiss).

## Figures and Tables

**Table 1:** KfrC_RA3_ interactants identified in *E. coli* DH5α and *A. veronii* library screenings.

Library	DNA Coordinates (Peptide) *	Gene	Predicted Function **	NCBI Accession Number	Number of Clones
***E. coli* DH5α**	792619 (209–332)	*edd*	phosphogluconate dehydratase	WP_001069467.1	1
	1321046 (266–446)	*fadL*	long-chain fatty acid transporter	WP_001295701.1	1
	2535141 (76–228)	*yhjJ*	Zn-dependent peptidase	WP_001163141.1	1
	3019820 (475–614)	*btuB*	vitamin B12 transporter	WP_000591359.1	1
	3869607 (138–272)	*cof*	HMP-PP phosphatase	WP_001336137.1	1
	4142310 (494–648)	*ybgQ*	outer membrane usher protein	WP_001350492.1	1
	4242142 (25–171)	*ompX*	outer membrane protein OmpX	WP_001295296.1	1
	**Most similar Protein (BLASTP)**				**Number of Clones**
***A. veronii***	**Protein Length (Peptide) §**	Predicted Function **		**NCBI Accession Number**	
	354 (103–354)	3-deoxy-7-phosphoheptulonate synthase		WP_113739212.1	1
	403 (184–403)	EAL domain-containing protein		WP_064340963.1	1
	385 (187–327)	acyl-CoA dehydrogenase		WP_129504156.1	1

*—position in the *E. coli* DH5α genome of the first nucleotide fused to *cyaA* fragment; amino acid residues of the fused polypeptides are indicated in brackets; **—potential function based on the comparison of protein domains, **§**—length of the *A. veronii* protein most similar to the fusion protein fragment; amino acid residues of the fused polypeptides are indicated in brackets.

**Table 2:** Screening of the RA3 library.

Bait	Coordinates *	Prey	Number of Clones	Cloned Fragment
**KfrC-T25**	16,448	VirD4	1	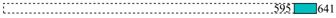
	16,451		8	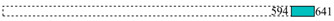
	16,511		1	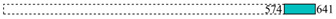
	16,931		3	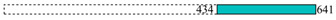
	17,633		1	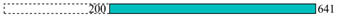
**T25-KfrC**	17,501	VirD4	2	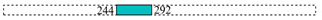
	17,549		1	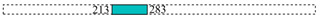
	24,447	VirB4	2	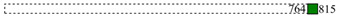
	4307	KfrC	3	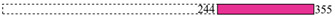

*—position of the first nucleotide of the fused fragment from the RA3 plasmid sequence.

**Table 3:** Plasmids used in this study.

Plasmids Provided by Others	
**Designation**	**Relevant Features or Description**
pABB19	*ori*_MB1_, Ap^r^, transcriptional terminator T*pro*/T*lyz* P1 [51]
pABB28.2	pET28a with *his*-tag replaced by *flag*-tag [52]
pAKB2.55	pGBT30 with *kfrC* without a stop codon (IBB) ^a^
pAKB2.70	pGBT30 with *kfrC*_RA3_*-yfp* (IBB) ^a^
pAKB7.5	*ori*_MB1_, Km^r^, *parS-oriT*_RA3_ (RA3 coordinates 9397–9854 nt) [24]
pAKB16.50	pLKB4 *cyaT18-korB*_RA3_ [17]
pAKB16.50N	pKGB4 *korB*_RA3_-*cyaT18* [17]
pAMB8	pBBR1MCS-3 modified in *tetM* to remove EcoRI site (IBB) ^a^
pBBR1MCS	IncA/C, Cm^r^, BHR cloning vector [53]
pBBR1MCS-2	IncA/C, Km^r^ BHR cloning vector [54]
pBGS18	*ori*_MB1_, Km^r^, cloning vector [55]
pESB5.58	pGBT30 with *tacp-kfrA* [18]
pET28a	*ori*_MB1_, Km^r^, T*7p*, *lacO*, His_6_-tag, T7 tag (Novagen)
pET28mod	pET28a derivative, T7 tag removed [56]
pGBT30	*oriV*_MB1_, Ap^r^, *lacI^q^*, *tacp* expression vector [57]
pJSB8.5.2	pLKB4 *cyaT18-virD4* (IBB) ^a^
pJSB9.5.1	pKGB4 *virD4-cyaT18* (IBB) ^a^
pJSB10.5.2	pLKB2 *cyaT25-virD4* (IBB) ^a^
pJSB11.5.1	pKGB5 *virD4-cyaT25* (IBB) ^a^
pKAB20	pUC19 derivative with *flag-mcs*^b^*-his_6_*; allows in-frame attachment of *flag* to 5′ and/or *his_6_* to the 3′ of a gene [58]
pKAB28	pET28mod with deletion of *his_6_-tag* and EcoRI site adjacent to RBS [57]
pKAB28.7	pET28mod derivative with *his_6_-* *mcs*^b^ [58]
pKD13	template plasmid for gene disruption [30]
pKD46	*ori*_R101_, *araB*p-*gam-bet-exo*, *repA*101(ts), Ap^r^, lambda Red recombinase expression plasmid [30]
pKGB4	*ori*_ColE1_, pUT18 with modified *mcs*, *lacp*- *mcs*^b^ -*cyaT18*, Ap^r^ (IBB) ^a^
pKGB5	*ori*_p15_, pKNT25 with modified *mcs*, *lacp*- *mcs*^b^ -*cyaT25*, Km^r^ (IBB) ^a^
pKT25-zip	pKT25 derivative encoding CyaT25 in translational fusion with leucine zipper of GCN4 [34]
pLKB2	*ori*_p15_, pKT25 with modified *mcs*, *lacp*-*cyaT25*- *mcs*^b^, Km^r^ [59]
pLKB4	*ori*_ColE1_, pUT18C with modified *mcs*, *lacp*-*cyaT18*- *mcs*^b^, Ap^r^ [59]
pMRA1.3	pLKB4 with *cyaT18-kfrC*_R751_ (IBB) ^a^
pMRB2.3	pKGB4 with *kfrC*_R751_*-cyaT18* (IBB) ^a^
pMRB3.3	pLKB2 with *cyaT25-kfrC*_R751_ (IBB) ^a^
pMRB4.3	pKGB5 with *kfrC*_R751_*-cyaT25* (IBB) ^a^
pOMB3.104	pUC18 derivative with *parS* P1 prophage (IBB) ^a^
pOMB4.13	pLKB4 with *cyaAT18*-*kfrA* [18]
pOMB4.15	pLKB4 with *cyaAT18*-*kfrC* [18]
pOMB5.13	pLKB2 with *cyaAT25*-*kfA* [18]
pOMB5.15	pLKB2 with *cyaAT25*-*kfrC* [18]
pOMB6.14	pKGB4 with *kfrA-cyaAT18* [18]
pOMB6.16.1	pKGB4 with *kfrC*-*cyaAT18* [18]
pOMB7.14	pKGB5 with *kfrA*-*cyaAT25* [18]
pOMB7.16.1	pKGB5 with *kfrC*-*cyaAT25* [18]
pOMB9.80	pGBT30 with *kfrA*_RA3_*-cfp* (IBB) ^a^
pUC18	*ori*_MB1_, Ap^r^, cloning vector [60]
pUT18C-zip	pUT18C derivative encoding CyaT18 in translational fusion with leucine zipper of GCN4 [34]
R751Tc^R^	IncPβ (IncP-1β) ^c^, Tc^r^-derivative of R751 [38]
RA3	IncU (IncP-6) ^c^, Cm^r^, Sm^r^, Su^r^ (F. Hayes)
RK2	IncPα (IncP-1α) ^c^, Ap^r^, Km^r^, Tc^r^ (C.M. Thomas)
**Plasmids Constructed during This Work**	
**Designation**	**Relevant Features or Description**
pESB5.88	pGBT30 with *tacp-kfrC*; annealed oligonucleotides 28 and 29 inserted between Xba-SalI of pAKB2.55
pESB5.90	pGBT30 with *tacp-kfrC* without a stop codon; annealed oligonucleotides 6 and 7 inserted between Xba-SalI of pAKB2.55
pESB10	pBBR1MCS-2 *lacI^q^ tacp* with transcriptional terminator T1/T2*_rrnB_*; PCR product obtained with primers 36 and 37 on *E. coli* genomic DNA inserted as XhoI-KpnI fragment between SalI-KpnI sites
pESB11	pOMB12.0 derivative with transcriptional terminator T1/T2*_rrnB_*; PCR fragment obtained with primers 36 and 37 on *E. coli* genomic DNA inserted between XhoI-KpnI sites
pESB11.58	pESB11 with *tacp*-*kfrA*; EcoRI-SalI fragment from pESB5.58 inserted between EcoRI-XhoI sites
pESB15	pET28a with annealed oligonucleotides 30 and 31 inserted between NcoI and BamHI sites
pESB15.90	pESB15 with *kfrC-his_6_*; EcoRI-HindIII fragment from pESB5.90
pJSB1.4	pBGS18 with the *mobCp-mobC-nic*; PCR fragment obtained with primers 26 and 5 on RA3 template inserted between EcoR-SalI sites (RA3 coordinates 9437–11355 nt)
pJSB1.5.2	pBGS18 with *virD4*; PCR fragment obtained with primers 44 and 45 on RA3 template cloned between the BamHI-KpnI sites (RA3 coordinates 18230–16305 nt)
pJSB1.8	pBGS18 with Tra_RA3_; pJSB1.4 with SmaI-SalI fragment of RA3 plasmid (RA3 coordinates 10733–22925 nt)
pJSB1.24	pBGS18 with Tra_RA3_-*korCp-korC*; PCR fragment *korCp-korC* obtained with primers 2 and 3 (RA3 coordinates 3093–3705) inserted into pJSB1.8
pJSB8.1	pLKB4 with *cyaT18-mobC*; PCR fragment obtained with primers 22 and 23, cloned between the EcoRI-HincII sites (RA3 coordinates 9837–10455 nt)
pJSB8.3	pLKB4 with *cyaT18-nic;* PCR fragment obtained with primers 25 and 26 cloned between the EcoRI-HincII sites (RA3 coordinates 10360–11355 nt)
pJSB8.5.2	pLKB4 with *cyaT18-virD4*; fragment BamHI-KpnI from pJSB1.5.2 cloned into pLKB4
pJSB8.35	pLKB4 with *cyaT18-mobC-oriT*_RA3_; SmaI-HincII fragment of pAKB7.5 carrying *parS-oriT* cloned into PvuII site of pJSB8.1
pJSB9.1.1	pKGB4 with *mobC-cyaT18*; PCR fragment obtained with primers 22 and 24 cloned between EcoRI-SacI sites, (RA3 coordinates 9837–10364 nt)
pJSB9.5.1	pKGB4 with *virD4-cyaT18*; PCR fragment obtained with primers 44 and 46 cloned between BamHI-SacI sites (RA3 coordinates 18230–16308 nt)
pJSB10.1	pLKB2 with *cyaT25-mobC*; PCR fragment EcoRI-HincII obtained with primers 22 and 23, cloned between the EcoRI-SmaI sites (RA3 coordinates 9837–10455 nt)
pJSB10.3	pLKB2 with *cyaT25-nic*; PCR fragment EcoRI-HincII obtained with primers 25 and 26 cloned between EcoRI-SmaI sites (RA3 coordinates10360–11355 nt)
pJSB10.5.2	pLKB2 with *cyaT25-virD4*; PCR fragment obtained with primers 44 and 45 cloned between BamHI-KpnI sites (RA3 coordinates18230–16305 nt)
pJSB11.1	pKGB5 with *mobC-cyaT25*; PCR fragment obtained with primers 22 and 24 cloned between EcoRI-SacI sites (RA3 coordinates 9837–10364 nt)
pJSB11.3	pKGB5 with *nic-cyaT25*; PCR fragment obtained with primers 25 and 27 cloned between EcoRI-SacI sites (RA3 coordinates 10360–11352 nt)
pJSB11.5.1	pKGB5 with *virD4-cyaT25*; PCR fragment obtained with primers 44 and 46 cloned between BamHI-SacI sites (RA3 coordinates 18230–16308 nt)
pOMB1.17	pBGS18 with *kfrC_1–249_*; PCR product amplified on RA3 template with primers 8 and 9 inserted between EcoRI-SalI sites (RA3 coordinates: 3692–4438)
pOMB1.18	pBGS18 with *kfrC_244–355_*; PCR product amplified on RA3 template with primers 10 and 11 inserted between EcoRI-SalI sites (RA3 coordinates: 4421–4756)
pOMB1.42	pBGS18 with *virD4_434–641_*; EcoRI-BamHI fragment from pOMB4.42
pOMB1.51	pBGS18 with *virD4_434–641_ kfrC*; PCR product amplified on RA3 template with primers 14 and 18 inserted as BglII-SalI fragment between BamHI-SalI sites of pOMB1.42 (RA3 coordinates: 3686–4756)
pOMB1.74	pBGS18 *virD4-cfp*; BamHI-HindIII fragment from pOMB9.80 with overhangs filled in using Klenow fragment of PolI inserted within EcoICRI site of pJSB1.5.2
pOMB2.0	pKAB20 derivative with Ecl136II restriction site inserted between MunI and HindIII sites (annealed oligonucleotides 33 and 34)
pOMB2.0.28	pUC19 with *kfrC_244__–__355_-his_6_*; EcoRI-SmaI fragment from pOMB1.18 inserted in EcoRI-Ecl136II sites of pOMB2.0
pOMB2.49	pUC19 with *flag*-*virD4*; PCR product amplified on RA3 template with primers 4 and 49 inserted between MunI-HindIII sites of pKAB20 (RA3 coordinates: 18230–16305)
pOMB2.50	pUC19 with *flag-vird4_434–641_*; EcoRI-SalI fragment from pOMB1.42 inserted between MunI-SalI sites of pKAB20
pOMB2.52	pUC19 with *flag-virD4_434–641_ kfrC-his_6_*; EcoRI-SalI fragment from pOMB1.51 inserted between MunI-XhoI sites of pKAB20
pOMB2.74	pUC19 *virD4-cfp*; PCR product amplified on pOMB1.74 template with primers 1 and 49 inserted as MunI-SmaI sites of pOMB2.0
pOMB4.0	pLKB4 derivative with I-SceI restriction site inserted into KpnI site (annealed oligonucleotides 20 and 21)
pOMB4.17	pLKB4 with *cyaT18-kfrC_1–249_*; EcoRI-SmaI fragment from pOMB1.17
pOMB4.18	pLKB4 with *cyaT18-kfrC_244–355_*; EcoRI-SmaI fragment from pOMB1.18
pOMB4.34	pLKB4 with *cyaT18-traG*_R751_; PCR product amplified on R751 template with primers 38 and 39 inserted as EcoRI-KpnI fragment (R751 coordinates: 48800–46887)
pOMB4.36	pLKB4 with *cyaT18-kfrC*_RK2_; PCR product amplified on RK2 template with primers 15 and 16 inserted as EcoRI-KpnI fragment (RK2 coordinates: 54424–53079)
pOMB4.38	pLKB4 with *cyaT18-traG*_RK2_; PCR product amplified on RK2 template with primers 40 and 41 inserted as EcoRI-KpnI fragment (RK2 coordinates: 48495–46588)
pOMB4.42	pLKB4 with *cyaT18*-*virD4_434–641_*; PCR product amplified on RA3 template with primers 47 and 48 inserted between EcoRI-BamHI sites (RA3 coordinates: 16931–16305)
pOMB5.17	pLKB2 with *cyaT25-kfrC_1–249_*; EcoRI-SmaI fragment from pOMB1.17
pOMB5.18	pLKB2 with *cyaT25-kfrC_244–355_*; EcoRI-SmaI fragment from pOMB1.18
pOMB5.34	pLKB2 with *cyaT25-traG*_R751_; EcoRI-KpnI fragment from pOMB4.34
pOMB5.36	pLKB2 with *cyaT25-kfrC*_RK2_; EcoRI-KpnI fragment from pOMB4.36
pOMB5.38	pLKB2 with *cyaT25-traG*_RK2_; EcoRI-KpnI fragment from pOMB4.38
pOMB6.17	pKGB4 with *kfrC_1–249_-cyaT18*; EcoRI-SmaI fragment from pOMB1.17
pOMB6.18	pKGB4 with *kfrC_244–355_-cyaT18*; EcoRI-SmaI fragment from pOMB1.18
pOMB6.35	pKGB4 with *traG*_R751_*-cyaT18*; PCR product amplified on R751 template with primers 38 and 43 inserted as EcoRI-SmaI fragment (R751 coordinates: 48800–46890)
pOMB6.37	pKGB4 with *kfrC*_RK2_*-cyaT18*; PCR product amplified on RK2 template with primers 15 and 17 inserted as EcoRI-SmaI fragment (RK2 coordinates: 54424–53082)
pOMB6.39	pKGB4 with *traG*_RK2_*-cyaT18*; PCR product amplified on RK2 template with primers 40 and 42 inserted as EcoRI-SmaI fragment (RK2 coordinates: 48495–46591)
pOMB7.17	pKGB5 with *kfrC_1–249_-cyaT25*; EcoRI-SmaI fragment from pOMB1.17
pOMB7.18	pKGB5 with *kfrC_244–355_-cyaT25*; EcoRI-SmaI fragment from pOMB1.18
pOMB7.35	pKGB5 with *traG*_R751_*-cyaT25*; EcoRI-SmaI fragment from pOMB6.35
pOMB7.37	pKGB5 with *kfrC*_RK2_*-cyaT25*; EcoRI-SmaI fragment from pOMB6.37
pOMB7.39	pKGB5 with *traG*_RK2_*-cyaT25*; EcoRI-SmaI fragment from pOMB6.39
pOMB8.28	pET28mod with *kfrC_244–355_-his_6_*; pKAB28 derivative with EcoRI-SalI fragment from pOMB2.0.28
pOMB8.49	pET28mod with *flag-virD4*; MunI-HindIII fragment from pOMB2.49 inserted between EcoRI-HindIII sites of pKAB28
pOMB8.50	pET28mod with *flag-virD4_434–641_*; pKAB28 derivative with EcoRI-SalI fragment from pOMB2.50
pOMB8.52	pET28mod with *flag-virD4_434–641_ kfrC-his_6_*; pKAB28 derivative with EcoRI-SalI fragment from pOMB2.52
pOMB9.18	pGBT30 with *tacp*-*kfrC_244–355_*; EcoRI-SalI fragment from pOMB1.18
pOMB9.29	pGBT30 with *tacp-kfrC-his_6_*; PCR product amplified on the pESB15.90 template with primers 19 and 35 inserted between XbaI-SalI sites of pESB5.88
pOMB9.31	pGBT30 with *tacp*-kfrC*; two-stage PCR was used for KfrC site-directed mutagenesis, described in detail in Metods, PCR final product was inserted between XbaI-SalI sites
pOMB12.0	pOMB12.30 derivative with transcriptional terminator T*pro*/T*lyz* P1; PCR product amplified on pABB19 as a template with primers 50 and 51 inserted as EcoRI-SalI fragment between EcoRI-XhoI sites
pOMB12.15	pESB11 with *tacp*-*kfrC*; EcoRI-SalI fragment from pESB5.88 inserted between EcoRI-XhoI sites
pOMB12.30	pBBR1MCS-3 *lacI^q^ tacp*; pAMB8 derivative with EcoRI-PstI fragment from pGBT30
pOMB12.74	pBBR1MCS-2 *virD4-cfp*; pESB10 derivative with MunI-SmaI fragment from pOMB2.74 inserted between EcoRI-SmaI sites
RA3Δ*incC*	*incC* gene replaced by Km^r^ cassette amplified on pKD13 template with primers 52 and 53 (coordinates of deletion: 6356–7080)
RA3Δ*nic*	*nic* gene replaced by Km^r^ cassette amplified on pKD13 template with primers 54 and 55 (coordinates of deletion: 10380–11352)
RA3Δ*virD4*	*virD4* replaced by Km^r^ cassette amplified on pKD13 template with primers 56 and 57 (coordinates of deletion: 18195–16314)
RA3Δ*parS*	*parS* site replaced by Km^r^ cassette amplified on pKD13 template with primers 58 and 59 (coordinates of deletion: 9707–9722)
RA3Δ*oriT*	*oriT* site replaced by Km^r^ cassette amplified on pKD13 template with primers 60 and 61 (coordinates of deletion: 9747–9756)
RA3*parS▼oriT*	Km^r^ cassette amplified on pKD13 template with primers 59 and 62 and inserted between *parS* and *oriT* sites (coordinates of insertion: 9722/9723)
RA3Δ*kfrA*	*kfrA* replaced by Km^r^ cassette amplified on pKD13 template with primers 63 and 64 (coordinates of deletion: 4892–5935)
RA3Δ*kfrC*	*kfrC* replaced by Km^r^ cassette amplified on pKD13 template with primers 65 and 66 (coordinates of deletion: 3695–4738)
RA3Δ(*kfrC-A*)	*kfrC*-*kfrA* replaced by Km^r^ cassette amplified on pKD13 template with primers 65 and 64 (coordinates of deletion: 3695–5935)
RA3#	*parS*_P1_-Km^r^ cassette inserted within integron at position 38,663 of RA3 genome

^a^—Institute of Biochemistry & Biophysics collection; ^b^—mcs, multiple-cloning site modified; ^c^—in brackets plasmid incompatibility groups in *Pseudomonas* spp.

**Table 4:** Oligonucleotides used in this study.

No	Designation	Sequence
1	CFPSmSaP	gccccggGGTCGAC**TTA**CTTGTACAGCTCG
2	CkorCD	cgacatgtTTATGTTCGG**TCA**TGGTTTC
3	CkorCG	gcgcatgcCTTAAAGGAGGTGCATAGGT
4	FLAGVirDR	ccaagctt**TTA**TGCCGCTTCAGCCAAGC
5	kasmob1	cggaattcacatgtTTCTCGTTGGAGGGTGATCA
6	KFRCBSD	tcgacaagcttCCGCT
7	KFRCBSG	CTAGAGCGGaagcttg
8	kfrCFL	gcaagctttggaattC**ATG**ACCGAACATAAGGCCGA
9	kfrCIR	cggtcgac**tta**cccgggAGCTCCGCTTTTGCCCATTC
10	kfrCIIF	cggaattc**ATG**GGCAAAAGCGGAGCTGA
11	kfrCIIR	cggtcgac**TTA**cccgggCCGCTCTAGATCGTCTTCAT
12	kfrCmutF	TGTCGcgGccgCGCTGGCGATGGGCG
13	kfrCmutR	CCAGCGcggCcgCGACAATCAGATAAGGCTGGTCA
14	kfrCrbsF	cggaattcagatcta*aggagG*AAACC**ATG**ACCGAACATAA
15	KfrCRK2N	gcgaattc**aTG**AGCAGCTACAGCAGAG
16	KfrCRK2R	gcggtacc**TTA**GCTGGGCTTGTTTGAC
17	KfrCRKst	cgcccgggGCTGGGCTTGTTTGACAGG
18	KfrCstop	cggtcgacCCGCTCTAGATCGTCTTCAT
19	KfrCXbaF	cgTCTAGAGCGGAAGCTTGCGG
20	LinkSceF	tagggataacagggtaatgtac
21	LinkSceR	attaccctgttatccctagtac
22	mobC1	cggaattc**ATG**GCAAAGAGCTATCGGATCG
23	mobC2	cggtcGACTCGCTTAACTCGGCCTTTCA
24	mobCT	gcgagctccTTCATCGATCCCCCACTTG
25	nic1	cggaattc**ATG**AATAAGGGCTATGACACTCTAGCCGGG
26	nic2	cggtcgac**TTA**TCTCTCGTCTTCGTCCC
27	Nic2k	gcgagctcgTCTCTCGTCTTCGTCCCTCTCTGATTTTGC
28	OKFRCD2	tcgacggtaccagcggct**tca**CCGCT
29	OKFRCG2	CTAGAGCGG**tga**agccgctggtaccg
30	OPETD	GATCGTGCAGC
31	OPETG	CATGGCTGCAC
32	pGBT30R	CTCTTCCGCATAAACGCTTC
33	podst4F	aattggggctcc
34	podst4R	agctggagctcc
35	T7TERR	gcgtcgacCAAAAAACCCCTCAAGACCC
36	TerpKKKF	cgcggtaccctcgagcccgggATCAGAACGCAGAAGCGGTC
37	TerpKKR	cgcggtaccagtactGGCTTGTAGATATGACGACAG
38	TraGEcoF	gcgaattc**ATG**AAGATCAAGATGAACAAC
39	TraGKpnR	gcggtacC**TCA**TATCGTGATGCCCTCCC
40	TraGRK2F	gcgaattc**ATG**AAGAACCGAAACAACGCC
41	TraGRK2R	gcggtacC**TCA**TATCGTGATCCCCTCC
42	TraGRKst	cgcccgggTATCGTGATCCCCTCCCCTTC
43	TraGSmaR	cgcccgggTATCGTGATGCCCTCCC
44	virD4Gm	gcggattc**ATG**ACCCAGAATTCAAACGGACAC
45	virD4Kpn	cgggtaCC**TTA**TGCCGCTTCAGCCAAGCCATT
46	virD4N	cggagctcCTGCCGCTTCAGCCAAGCCATTAA
47	VirDfr2F	gcgaattcTTGCGTGAAACATATGGG
48	VirDfrBR	gcggaTCC**TTA**TGCCGCTTCAGCCAAG
49	VirDMunF	cgcaattg**ATG**ACCCAGAATTCAAACG
50	TProLyzF	gcgaattctacgtactcgagagatctACATGTGGTACCAACCACC
51	TProLyzR	gcgtcgacCCATGGATAATAGTTAACGAG
52	delincF	*CGAGGATGAGGCATATAAACAGGCTAATAAACCAAAGGGT**TGA**GCAT**ATG***ATTCCGGGGATCCGTCGACC
53	delincR	*CCGCTGAGGTCTGCCCCTTTACCAC**TCAT**TCAGCCACCCCCATTTTTTCA*TGTAGGCTGGAGCTGCTTCG
54	delnicF	*TCGCCGGTTTGCTTCAACGCAACTTAAACAAGTGGGGGATCG**ATG**AA**TA******A***ATTCCGGGGATCCGTCGACC
55	delnicR	*GAACGC**TAA**ATACCTGAAAACAAAAACCGGCCAACAGGCCGGTTTTT**TTA***TGTAGGCTGGAGCTGCTTCG
56	delvirF	*TAACGGAGATTTACT**ATG**ACCCAGAATTCAAACGGACACAAATGGCG**TA******A***ATTCCGGGGATCCGTCGACC
57	delvirR	*TATGTTTTTTCCTGTGCAATATTTGC**CAT**TTCAATTATTCC**TTA**TGCCGC*TGTAGGCTGGAGCTGCTTCG
58	delparF	*CGACCTGGTGAGCCTGGCCGAAGGCCAAAAGCCACTGCAAAACCGAAAAA*ATTCCGGGGATCCGTCGACC
59	delparR	*AGACGCTAGCAAATTGCGAATCCTGCCCTAGTTCTAACCCCCCCATGTTT*TGTAGGCTGGAGCTGCTTCG
60	deloriF	*AACCGAAAAATTTCGTACGTACGAAAAAACATGGGGGGGTTAGAACTAGG*ATTCCGGGGATCCGTCGACC
61	deloriR	*GGGGGACAGGTGCAATTTTAGCACAAGCGGCGGCAGACGCTAGCAAATTG*TGTAGGCTGGAGCTGCTTCG
62	oriparF	*GCCGAAGGCCAAAAGCCACTGCAAAACCGAAAAATTTCGTACGTACGAAA*ATTCCGGGGATCCGTCGACC
63	delkfrAF	*ATGTATTGTATTAAAATACAATACATACAATACAGGGAGCCGAAGCC**ATG***TGTAGGCTGGAGCTGCTTCG
64	delkfrAR	*CACTTTATCTGTTTACGTCAATAGATAGGGG**TTA**CTCTTTGGTGTCGGCTGC*ATGGGAATTAGCCATGG
65	delkfrCF	*CCTGGCAGGTTTCGGGGCTATATGGGACGC**TGA**CCGGGATTGAAACC**ATG***TGTAGGCTGGAGCTGCTTCG
66	delkfrCR	*AATGGCCGGGGTCGGTGACAGGGTAGCGGCTTCACCGCTCTAGATCGTCTTC*ATGGGAATTAGCCATGG
67	Kmpar1F	*GGTGCAAAGACGCCGTGGAAGCGTGTGAGGTTGACTCGCGGCTTAGGTAC*ATTCCGGGGATCCGTCGACC
68	Kmpar2R	TGTAGGCTGGAGCTGCTTCG
69	Kmpar3F	cgaacgagctccagcctacaCTTGCATGCCTGCAGGGTAC
70	Kmpar4R	*CCCATGTGATCTTCGAGCCGCTGGACTTCATCGCCAAACTCGCTGCGTTG*GGTACCCTGCCGGGGTTCTC

Start and stop codons are marked in bold, the introduced restriction sites or overhangs are underlined, nucleotides not complementary to the template are shown in small letters, an additional Shine–Dalgarno sequence is underlined, and oligonucleotides used for mutagenesis [30] sequence corresponding to RA3 is in italics.

## Data Availability

All obtained data is included in the manuscript and Supplement.

## Supplementary Materials

The following are available online at https://www.mdpi.com/article/10.3390/ijms22094880/s1, Figure S1: Determination of the *A. veronii* genomic DNA fragments mean size and insertion efficiency into pUT18C derivative vector—colony PCR products visualized on an agarose gel, Figure S2: Validation of the KfrC-T25 interactions found in the (A) *E. coli* DH5α, (B) *A. veronii* and (C) RA3 genome-fragment libraries screening, Figure S3: Alignment of VirD4_RA3_ (ABD64846) and TraG_RK2_ (Q00184).
