# Supplementary material for: Alpha-Helical Protein KfrC Acts as a Switch between the Lateral and Vertical Modes of Dissemination of Broad-Host-Range RA3 Plasmid from IncU (IncP-6) Incompatibility Group
Source: Int J Mol Sci. 2021 May 5;22(9):4880. doi: 10.3390/ijms22094880 (PMC8124265; doi:10.3390/ijms22094880)
Supplement: Supplementary file 1 [file ijms-22-04880-s001.zip › ijms-1186255-supplementary.pdf]

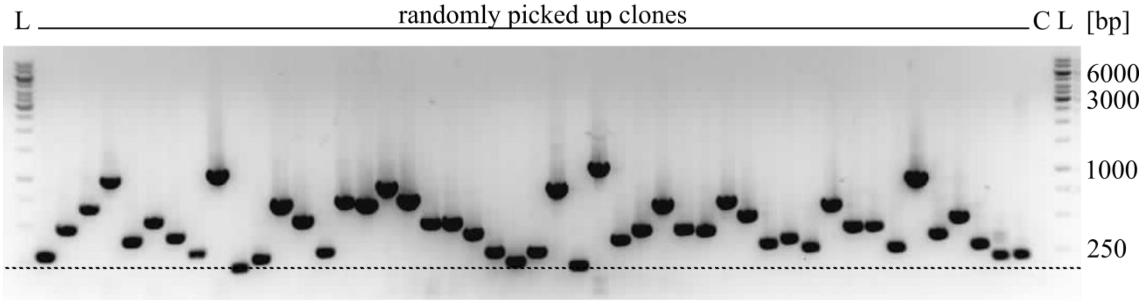

Figure S1. Determination of the *A. veronii* genomic DNA fragments mean size and insertion efficiency into pUT18C derivative vector – colony PCR products visualized on an agarose gel. Flanking lanes L – Gene Ruler 1kb DNA ladder; lane C – negative control, PCR without DNA. The size of the PCR product for the empty vector is indicated by a black dotted line.

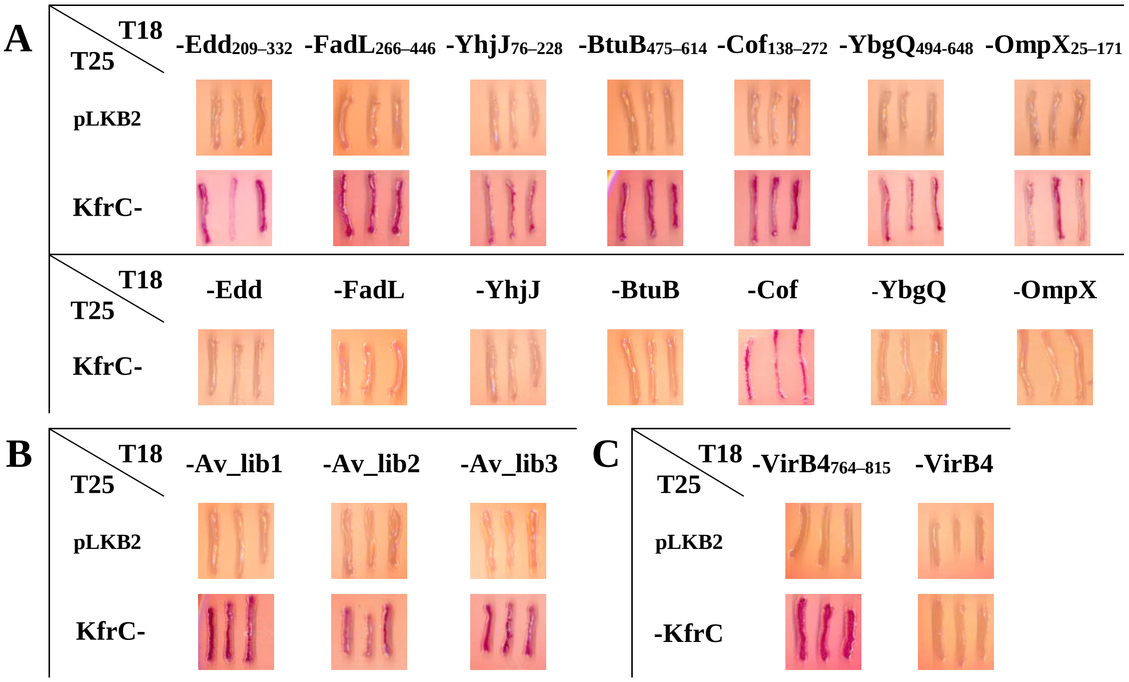

Figure S2. Validation of the KfrC-T25 interactions found in the (A) *E. coli* DH5α, (B) *A. veronii* and (C) RA3 genome-fragment libraries screening. *E. coli* BTH101 cells harbouring either pOMB7.16.1 (*kfrC-cyaT25*), pOMB5.15 (*cyaT25-kfrC*) or empty pLKB2 vector were transformed with pOMB4.0 derivatives encoding truncated or full-length forms of potential KfrC interactants.

VirD4$_{RA3}$ 1 MTQNSNGHKW RKYAILFGVP LFILAWVWLA GAIFMAGNGL NAKEATPLTL YQYWYYYGDI
TraG$_{RK2}$ 1 MKIKMNNAVG PQVRTAKPKP SKLLPVLGAA SMVGGLQAAT QFFAHTFAYH ATLGPNVGHV

VirD4$_{RA3}$ 61 ---------- ---------K KTQAWITGAA FGSLLILLLP VFLYFMPKK- ---ESLFGDA
TraG$_{RK2}$ 61 YAPWSILHWT YKWYSQYPDE IMKAGSMGML VSTVGLLGVA VAKVVTSNSS KANEYLHGSA

VirD4$_{RA3}$ 98 RWATEKEIKD SGLYS-EDGI --IVGVKTA- ------FFGL IKK-----YL IFGGAQHVLM
TraG$_{RK2}$ 121 RWAEKKDIQA AGLLPRERNV LEIVTGKAAP TATGVYVGGW QDKDGNFFYL RHSGPEHVLT

VirD4$_{RA3}$ 143 AAPTRSGKGV SIVIPNLLSW KDSVVVLDIK QENWDITSGF RAKHGQECYL LNLAPRDYRS
TraG$_{RK2}$ 181 YAPTRSGKGV GLVVPTLLSW GASSVITDLK GELWALTAGW RQKHAKNKVL RFEPASTSGG

VirD4$_{RA3}$ 203 HRWNPLFYIS DDPNFRINDI QKIGQMLF-P KVENEAPIWQ SS**ARSLWLGL VL---YLIET**
TraG$_{RK2}$ 241 VCWNPLDEIR LGTEYEVGDV QNLATLIVDP DGKGLDSHWQ KTAFALLVGV ILHALYKAKD

VirD4$_{RA3}$ 259 **EELPVTMGEA LRQLTMGDER LAEI-V**E--Q RQESDNPLSD ECYLALKEYL DTPDKTRGSV
TraG$_{RK2}$ 301 DGGTATLPSV DAMLADPNRD IGELWMEMAT YGHVDGQNHH AIGSAARDMM DRPEEEAGSV

VirD4$_{RA3}$ 316 RKGFTASLEL FYNPVIDAAT SGNDFDLRDL RKRR--MSVY VGITPDDLDR LAPLINLFFQ
TraG$_{RK2}$ 361 LSTAKSYLAL YRDPVVARNV SRSDFRIKQL MHEDDPVSLY IVTQPNDKAR LRPLVRVMVN

VirD4$_{RA3}$ 374 QVIDLNTREL PEQN----PD LKHSCLLLMD EFTAMGKVGI LSKGISYIAG YGLRMLPIIQ
TraG$_{RK2}$ 421 MIVRLLADKM DFEGGRPVAH YKHRLLMMLD EFPSLGKLEI MQESLAFVAG YGIKCYLICQ

VirD4$_{RA3}$ 430 SPAQL--RET -YGADAAETF IDNHALQIVF APKNIKVAKE ISDSLGTKTV -KNKS-----
TraG$_{RK2}$ 481 DINQLKSRET GYGHD--ESI TSNCHVQNAY PPNRVETAEH LSRLTGQTTV VKEQITTSGR

VirD4$_{RA3}$ 481 RSRQLTGKTS RSENASDTGR ALLMPQEVKQ I--------G QKAE----IL LLENCPPIKC
TraG$_{RK2}$ 539 RTAAMLGQVS RTYQ--EVQR PLLTPDECLR MPGPKKNAQG EIEEAGDMVI YVAGYPAIYG

VirD4$_{RA3}$ 529 SKITWYADQT FNERGNGRDG VKFPSPAVPL VDPNNRPKGE VSFHSNKIED AETSEKTVVE
TraG$_{RK2}$ 597 KQPLYFKDPV FSAR------ AAIPAPKVSD RLRAVAQADT EGEGITI 637

VirD4$_{RA3}$ 589 RDITVA**DIEN IDNLNLDDFS CDFSKIEIPE GSISDDAMDD LVSQFFNGLA EAA** 641

Figure S3. Alignment of VirD4$_{RA3}$ (ABD64846) and TraGRK2 (Q00184). Similar residues are shaded. Two VirD4 fragments found interacting with KfrC are in bold.

1. *Detailed description of genome-wide library construction of* E. coli, A. veronii *and RA3 plasmid using BACTH system*

Isolated genomic DNAs of *E. coli* DH5α, *A. veronii* or RA3 plasmid DNA were randomly fragmented by sonication on ice. Sonication conditions were optimized and determined experimentally for each sample via DNA visualization on agarose gel to get fragments ranging in size from 250 to 1500 bp. In the next step, the concentration of DNA was determined and an appropriate amount of DNA was used for the blunt ending of single-stranded overhangs with Fast DNA End Repair Kit, followed by purification with the usage of DNA Clean Up kit. In parallel pOMB4.0, pUT18C derivative modified to contain I-SceI restrictions site within the multiple cloning site (MCS), was digested using SmaI and Ecl136II restriction enzymes, dephosphorylated with FastAP Thermosensitive Alkaline Phosphatase and purified. Finally, obtained genomic DNA fragments were ligated with linear pOMB4.0. Ligation mixtures were re-digested using I-SceI to linearize the empty vectors and numerous electrotransformations of *E. coli* DH5α strain were performed. The mean number of grown colonies after each electrotransformation step was estimated based on plates with appropriate transformation mixture dilutions. All bacteria present at the surface of the selective plates were collected and plasmid DNA extraction using Plasmid Midi kit was performed. Quality of obtained genomic library was evaluated as described previously [62].

2. *Detailed description of Western blot analysis*

Nitrocellulose membrane with electrotransferred proteins was incubated in TBST buffer (20 mM Tris-HCl pH 7.5, 150 mM NaCl, 0.05% Tween 200) with 5% skim milk. After two hours the blocked membrane was incubated in TBST buffer with 5% skim milk containing 1:2000 diluted appropriate primary antibodies (Invitrogen) for two hours at room temperature. Next, the membrane was washed three times for 10 minutes in TBST buffer. Washed membrane was then incubated with TBST buffer with 5% skim milk containing 1:10000 diluted secondary antibodies conjugated with alkaline phosphatase (Promega) for one hour at room temperature. The two 5 minutes washes in TBST buffer and one in 10 ml of AP buffer (0.1 M Tris-HCl pH 9.5, 100 mM NaCl, 5 mM $MgCl_2$) were performed and the development step was carried out in the dark with AP buffer containing BCIP (5-bromo-4-chloro-3-indolyl-phosphate) and NBT (nitro blue tetrazolium) Color Development Substrate prepared according to the manufacturer's instructions (Promega). Depending on the signal strength the development step was performed from a few minutes to 12 hours in the dark. The reaction was stopped by rinsing the membranes with water.

3. *Detailed description of co-immunoprecipitation of KfrC with VirD4*

The formaldehyde-treated cells harvested by centrifugation for 10 minutes at 5000xg at 4°C, washed twice with 10 ml of ice-cold PBS buffer (15 mM KCl, 150 mM NaCl and 10 mM NaPi, pH 7.4) and resuspended in 0.5 ml of the lysis buffer [10 mM Tris-HCl (pH 8.0), 20% (w/v) sucrose] with lysozyme (1 mg/ml). After 30 minutes of incubation on ice, 200 µl of IP buffer [1.5 M Tris-HCl (pH 7.0), 0.3 M NaCl, 0.2% (v/v) Triton X-100], 1 mM protease inhibitor cocktail (Sigma) were added and the samples were incubated on ice for 10 minutes and then 5 minutes at room temperature. Cells were disrupted by sonication and the cell extracts were centrifuged twice at 15000xg for 20 minutes at 4°C. Cleared extracts (20-120 µl) were added to the mixture of immunoprecipitation buffer and lysis buffer in proportion 2:5 supplemented with 5 µg of anti-His antibodies (Invitrogen), 1 U of viscolase (A&A Biotechnology) and 10 mM $MgCl_2$, in a final volume of 500 µl. Samples were incubated overnight at 4°C with gentle agitation. Subsequently, 50 µl of A-sepharose (GE Healthcare) were added and after 2 hours of incubation at room temperature on rotating shaker suspensions were washed 8 times with the lysis buffer. The proteins content was analyzed by Western blotting.
